# Supplementary material for: Health behaviours and mental and physical health status in older adults with a history of homelessness: a cross-sectional population-based study in England
Source: BMJ Open. 2019 Jun 14;9(6):e028003. doi: 10.1136/bmjopen-2018-028003 (PMC6585828; doi:10.1136/bmjopen-2018-028003)
Supplement: Author's manuscript [file bmjopen-2018-028003.draft_revisions.pdf]

BMJ Open

BMJ Open is committed to open peer review. As part of this commitment we make the peer review history of every article we publish publicly available.

When an article is published we post the peer reviewers' comments and the authors' responses online. We also post the versions of the paper that were used during peer review. These are the versions that the peer review comments apply to.

The versions of the paper that follow are the versions that were submitted during the peer review process. They are not the versions of record or the final published versions. They should not be cited or distributed as the published version of this manuscript.

BMJ Open is an open access journal and the full, final, typeset and author-corrected version of record of the manuscript is available on our site with no access controls, subscription charges or pay-per-view fees (http://bmjopen.bmj.com).

If you have any questions on BMJ Open's open peer review process please email info.bmjopen@bmj.com

BMJ Open

Health behaviours and mental and physical health status in older adults with a history of homelessness: a population-based study

Journal:	*BMJ Open*
Manuscript ID	bmjopen-2018-028003
Article Type:	Research
Date Submitted by the Author:	19-Nov-2018

Complete List of Authors:	Smith, Lee; Anglia Ruskin University, Department of Life Sciences<br>Veronese, Nicola; National Research Council, Neuroscience Institute, Aging Branch<br>López-Sánchez, Guillermo Felipe; Faculty of Sport Sciences, University of Murcia<br>Moller, Eloise ; The Single Homeless Project<br>Johnstone, James ; The Cambridge Centre for Sport and Exercise Sciences, Anglia Ruskin University<br>Firth, Joseph ; NICM Health Research Institute, Western Sydney University<br>Grabovac, Igor; Centre for Public Health, Medical University of Vienna, Department of Social and Preventive Medicine<br>Yang, Lin; Medical University of Vienna, Epidemiology<br>Soysal, Pinar; Faculty of Medicine, Bezmialem Vakif University, Department of Geriatric Medicine<br>Jackson, Sarah ; University College London, Department of Behavioural Science and Health

Keywords:	Homelessness, physical health, MENTAL HEALTH, health behaviour, older adults

SCHOLARONE™
Manuscripts

Health behaviours and mental and physical health status in older adults with a history of homelessness: a population-based study

Smith L,[1*] Veronese N,[2] López-Sánchez GF, [3] Moller E,[4] Johnstone J,[5] Firth J,[6] Grabovac I,[7] Yang L,[8] Soysal P,[9] Jackson SE[10*]

1. The Cambridge Centre for Sport and Exercise Sciences, Anglia Ruskin University, Cambridge. Lee.Smith@anglia.ac.uk

2. National Research Council, Neuroscience Institute, Aging Branch, Padova, Italy. ilmannato@gmail.com

3. Faculty of Sport Sciences, University of Murcia, Spain. gfls@um.es

4. The Single Homeless Project, UK, London. Emoller@shp.org.uk

5. The Cambridge Centre for Sport and Exercise Sciences, Anglia Ruskin University, Cambridge. james.johnstone@anglia.ac.uk

6. NICM Health Research Institute, Western Sydney University, Westmead, Australia. j.firth@westernsydney.edu.au

7. Department of Social and Preventive Medicine, Centre for Public Health, Medical University of Vienna, Vienna, Austria. igor.grabovac@meduniwien.ac.at

8. Department of Epidemiology, Center for Public Health, Medical University of Vienna, Vienna, Austria. Lin.yang@muv.ac.at

9. Department of Geriatric Medicine, Faculty of Medicine, Bezmialem Vakif University, Istanbul, Turkey. dr.pinarsoysal@hotmail.com

10. Department of Behavioural Science and Health, University College London, London, UK. s.e.jackson@ucl.ac.uk

***Corresponding authors:** Dr Sarah E Jackson, PhD. Department of Behavioural Science and Health, University College London, 1-19 Torrington Place, London WC1E 6BT s.e.jackson@ucl.ac.uk; and Dr Lee Smith, PhD. Cambridge Centre for Sports and Exercise Sciences, Anglia Ruskin University, Cambridge lee.smith@anglia.ac.uk

Word count (excluding title page, abstract, references, figures and tables): 2726.

For peer review only - http://bmjopen.bmj.com/site/about/guidelines.xhtml

For peer review only

Health behaviours and mental and physical health status in older adults with a history of homelessness: a population-based study

ABSTRACT

Objectives: This study compared (i) levels of engagement in lifestyle risk behaviours and (ii) mental and physical health status in individuals who have previously been homeless to those of individuals who have not. **Design:** Cross-sectional. **Participants:** Data were from participants (n=6,931) of the English Longitudinal Study of Ageing. **Measures:** Participants reported whether they had ever been homeless. We used regression models to analyse associations between homelessness and (i) cigarette smoking, daily alcohol consumption and physical inactivity, adjusting for sociodemographic covariates (age, sex, ethnicity, highest level of education, marital status, and household non-pension wealth), and (ii) self-rated health, limiting long-standing illness, depressive symptoms, life satisfaction, quality of life and loneliness, adjusting for sociodemographics and health behaviours. **Results:** 104 participants (1.5%) reported having been homeless. Individuals who had been homeless were significantly more likely to smoke (OR=1.78, 95% CI 1.14 to 2.78), have a limiting long-standing illness (OR=2.49, 95% CI 1.53 to 4.03) and be depressed (OR=3.30, 95% CI 2.01 to 5.42), and scored lower on measures of life satisfaction (17.34 vs. 19.96, $p<0.001$) and quality of life (39.02 vs. 41.21, $p=0.013$). Rates of daily drinking (27.6% vs. 22.8%, $p=0.385$), physical inactivity (30.7% vs. 23.0%, $p=0.345$), poor self-rated health (41.9% vs. 30.5%, $p=0.050$) and loneliness (27.1% vs. 21.0%, $p=0.080$) were also elevated. **Conclusions:** Those who were once homeless have poorer mental and physical health outcomes and are more likely to engage in lifestyle risk behaviours. Interventions to improve their health and quality of life are required.

Keywords: Homelessness, physical health, mental health, health behaviour, older adults.

For peer review only - http://bmjopen.bmj.com/site/about/guidelines.xhtml

Strengths and limitations of this study

- To our knowledge, this is the first study to examine health outcomes in those who have transitioned out of homelessness.
- The very small number of participants with a history of homelessness in our sample meant that analyses were underpowered to detect modest differences between groups.
- However, the fact that we observed significant differences in the majority of the outcomes we analysed attests to the strength of these associations.
- Information on time since the period(s) of homelessness was not available so we were unable to evaluate the extent to which recency of homelessness is related to our outcomes of interest.
- It is possible that participants who reported a history of homelessness had transitioned out of homelessness many years or even decades prior. If this is the case, it demonstrates the long-lasting impact of homelessness across the lifespan.

For peer review only

For peer review only - http://bmjopen.bmj.com/site/about/guidelines.xhtml

INTRODUCTION

Homelessness is a substantial and growing problem in the United Kingdom. The annual homelessness monitor from Crisis and the Joseph Rowntree Foundation showed that in 2015/16 there were 271,000 local authority homelessness case actions in the UK, a rise of 32% since 2009/10.[1] Being homeless, or at risk of homelessness, has been shown to have a detrimental impact upon mental and physical health.[2]A recent systematic review concluded that people who are homeless are at increased risk of respiratory conditions, depression, anxiety, and excess winter mortality, compared to the general population.[3] Homelessness is associated with premature death, with the single homeless at the highest risk with an average age of death at 47 years, some 30 years lower than in the general population.[4]

Increased morbidity and mortality among the homeless may be driven, at least in part, by higher levels of engagement in lifestyle risk behaviours. Data from the USA indicate that while 19.8% of adult Americans smoke, smoking prevalence is over 70% among those who are homeless.[5–8] Levels of physical activity are also low among the homeless. In a Danish study, approximately 70% of homeless individuals reported no participation in any form of exercise.[9] High levels of alcohol consumption and drug use are also common among this population.[10]

Tackling homelessness is an urgent priority, and targeted policies have been actioned in the UK to rehouse those who are homeless. The Homelessness Directorate was established in 2002 in order to assist local authorities in tackling homelessness.[11] Strategies focus on preventing the need for people to sleep rough in the first place, as well as supporting people to move on from homelessness by helping them to address their needs, improving access to health and substance misuse services, and helping them rebuild their lives through education, training and employment.[12] A number of UK charities (e.g. Crisis, Shelter England, The Single Homeless Project) also work to support people who are homeless in acquiring a home and entering back into employment. With such policies and charities in operation, a significant number of individuals are able to transition out of homelessness.

While the evidence base on the health risks associated with homelessness is growing, to our knowledge no studies have explored what happens to the health and wellbeing of people when they are no longer

For peer review only - http://bmjopen.bmj.com/site/about/guidelines.xhtml

homeless. Given that lifestyle behaviours tend to track over the life course,[13] and early life exposures can have a substantial impact on later-life health outcomes,[14] it seems likely that the health risks associated with homelessness may persist, at least to some extent, beyond the period of homelessness. The present study aimed to investigate this through a comparison of (i) levels of engagement in lifestyle risk behaviours and (ii) mental and physical health status in individuals who have previously spent a period of time in their lives as homeless to those of individuals who have never been homeless, in a population-based sample of older adults living in England.

METHODS

Study population

Data were from the English Longitudinal Study of Ageing (ELSA), a nationally-representative longitudinal panel study of men and women aged 50 and older living in households across England.[15] The study began in 2002, with subsequent rounds of data collection at two-year intervals via computer-assisted personal interview and self-completion questionnaires. Wave 3 (2006/07) included a life history questionnaire, which gathered detailed information about important events that occurred in the participants' lives, including whether they had ever been homeless. Of the 9,771 participants interviewed in Wave 3 of ELSA, 7,855 (80.4%) completed the life history questionnaire. We excluded 924 participants (11.8%) with missing data on homelessness or sociodemographic covariates, leaving a final sample for analysis of 6,931 men and women. Ethical approval was obtained from the National Research Ethics Service and all participants gave full informed consent.

Patient and Public Involvement

Patients were not involved in the design of this study.

Measures

History of homelessness

Participants were asked whether they had ever been homeless for one month or more (yes/no).

Health behaviours

For peer review only

Smoking status was assessed with the question "*Do you smoke cigarettes at all nowadays*" (yes/no).

Frequency of alcohol intake over the past 12 months was reported on an 8-point scale from "not at all in the last 12 months" to "almost every day". We dichotomised responses to distinguish between participants drinking almost every day ("daily drinking") vs. less than this.

Physical activity was assessed with three items that asked respondents about the frequency with which they took part in vigorous, moderate and low-intensity activities (more than once a week, once a week, 1-3 times a month, hardly ever/never)[16]. Responses were dichotomised as follows: inactive (no moderate/vigorous activity on a weekly basis) vs. active (moderate or vigorous activity at least once a week).

Health and wellbeing

Self-rated health was assessed using a single item: *"Would you say your health is… very good/good/fair/bad/very bad?"*We analysed the proportion of individuals rating their health as fair, bad or very bad, as is commonly done in analyses of this variable.[17,18]

Limiting long-standing illness was assessed with two questions: (1) "*Do you have any long-standing illness, disability, or infirmity? By long-standing I mean anything that has troubled you over a period of time or that is likely to affect you over a period of time."* If they responded yes, they were asked (2) *"Does this illness or disability limit your activities in any way?"* Affirmation of a long-standing illness and any form of limitation classified the participant as having a limiting long-standing illness.

Depressive symptoms were assessed with an eight-item version of the Center for Epidemiologic Studies Depression Scale (CES-D) [19], a scale highly validated for use in older adults [20]. This asks about feelings over the last week (e.g. "Over the last week have you felt sad"), with binary response options (1=yes, 0=no). Positively framed items were reverse scored. Data were dichotomised using an established cut-off, with a score of 4 or higher indicating significant symptomatology.[20]

Life satisfaction was assessed with the Satisfaction With Life Scale,[21] which asks respondents to rate the extent to which they agree with five statements: "*In most ways my life is close to my ideal*"; "*The conditions of my life are excellent*"; "*I am satisfied with my life*"; "*So far I have got the important things I want in life*"; "*If I could live my life again, I would change almost nothing"* on a scale from 0 (strongly disagree) to 6

For peer review only - http://bmjopen.bmj.com/site/about/guidelines.xhtml

(strongly agree). Responses are summed to produce a total score between 0 and 30, with higher scores indicating greater life satisfaction.

Quality of life was assessed with the CASP-19,[22] a scale designed to measure quality of life in older people. Items cover four domains of quality of life; control (e.g. "*I feel that what happens to me is out of my control*"), autonomy (e.g. "*My health stops me from doing things I want to do*"), self-realisation (e.g. "*I feel that life is full of opportunities*"), and pleasure (e.g. "*I enjoy being in the company of others*"). Respondents are asked how often each statement applies to them (often=0, sometimes=1, not often=2, never=3). Positively-worded items are reverse scored so that a higher total score indicates higher quality of life (range: 0–57).

Loneliness was measured using a three-item short form of the Revised University of California Los Angeles (UCLA) Loneliness Scale.[23] Participants were asked: "*How often do you feel you lack companionship?*"(hardly ever or never=1, some of the time=2, often=3). Scores ranged from 3 to 9, with higher scores indicating greater loneliness. They were dichotomised at ≥6 versus <6 to indicate high versus low loneliness.[24]

Sociodemographic covariates

Participants reported their age, sex, ethnicity (white/non-white), highest level of education (no qualifications/up to degree/degree or higher), marital status (married/unmarried). Socioeconomic status was indexed by household non-pension wealth, which has been identified as a particularly sensitive indicator in this population).[25] Data on wealth were analysed as quintiles calculated across the whole Wave 3 ELSA sample.

Interviewers collected information on age, sex, ethnicity, the highest level of education, marital status and wealth. For these analyses, ethnicity was categorised as white or non-white. We classified education as low (no formal qualifications), intermediate (up to degree) or high (degree or higher). Marital status was categorised as married or unmarried (never married, divorced or widowed).Total wealth (excluding regular pension payments but including lump sums from private pension that have already been received but not yet consumed) was defined as financial wealth, physical wealth (such as business wealth, land or jewels) and housing wealth (primary and secondary residential housing wealth) minus debts. Wealth was categorised into five equal groups of net total non-pension wealth measured at the benefit unit level (a benefit unit is a couple or single person along with any dependent children they might have) across all ELSA

For peer review only

For peer review only - http://bmjopen.bmj.com/site/about/guidelines.xhtml

participants who took part in Wave 3. Wealth has been identified as a particularly appropriate indicator of SES in this age group.[25]

Statistical analysis

All analyses were conducted using SPSS version 24. Data were weighted to correct for sampling probabilities and for differential non-response and to calibrate back to the 2011 National Census population distributions for age and sex. The weights accounted for the differential probability of being included in Wave 3 of ELSA and for non-response to the life history interview.

Differences in sociodemographic characteristics of the groups who did and did not report a history of homelessness were tested using independent t-tests for continuous variables and chi-square tests for categorical variables. We used binary logistic regression to analyse associations between history of homelessness and cigarette smoking, daily drinking and physical inactivity, adjusting for sociodemographic covariates. We then used linear regression (for continuous outcomes) and binary logistic regression (for categorical outcomes) to analyse associations between history of homelessness and health and wellbeing, adjusting for sociodemographics and health behaviours. In all models, the reference category was the group without a history of homelessness. A *p*-value <0.05 was used to indicate statistical significance.

RESULTS

Sample characteristics

Of the 6,931 participants in our sample, 104 (1.5%) reported having been homeless for one month or more and 6,827 (98.5%) had never been homeless for one month or more. Sample characteristics in relation to history of homelessness are summarised in Table 1. On average, participants who had been homeless were significantly younger than those who had not been homeless (60.9 vs. 65.7 years) and a greater proportion were non-white (6.6% vs. 3.1%), unmarried (54.3% vs. 33.8%) and from the lowest quintile of wealth (44.3% vs. 18.9%). A marginally higher proportion of the group who had been homeless were male (53.8% vs. 46.7%) although the difference was not statistically significant (*p*=0.149). There was no significant difference between groups in the highest level of education achieved.

Table 1 Sample characteristics in relation to history of homelessness

	Had not been	Had been

For peer review only - http://bmjopen.bmj.com/site/about/guidelines.xhtml

For peer review only

	homeless (*n*=6,827)[1]	homeless (*n*=104)	*p*
Age (years), mean (SD)	65.74 (10.64)	60.94 (8.32)	<0.001
Sex			
Men	46.7	53.8	0.149
Women	53.3	46.2	-
Ethnicity			
White	96.9	93.4	0.042
Non-white	3.1	6.6	-
Highest level of education			
No qualifications	32.4	27.4	0.541
Below degree	52.2	56.6	-
Degree or higher	15.5	16.0	-
Marital status			
Married	66.2	45.7	<0.001
Unmarried	33.8	54.3	-
Wealth quintile			
1 (poorest)	18.9	44.3	<0.001
2	19.5	13.2	-
3	20.6	14.2	-
4	20.0	16.0	-
5 (richest)	21.0	12.3	-

[1] Unweighted sample sizes.
All figures are weighted for sampling probabilities and differential non-response.
Values are percentages unless otherwise stated.
SD = standard deviation.

History of homelessness and health behaviours

Associations between history of homelessness and health behaviours are shown in Table 2. After adjustment for age, sex, ethnicity, education, marital status and wealth, participants who had been homeless had 1.78 times higher odds (95% CI 1.14 to 2.78) of being a smoker than those who had not been homeless (20.2% vs. 15.4%, *p*=0.011). Rates of daily drinking (27.6% vs. 22.8%) and physical inactivity (30.7% vs. 23.0%) were also higher in the group who had been homeless, but differences were not statistically significant.

Table 2 Associations between history of homelessness and health behaviours

	Had not been homeless	Had been homeless	*p*
Smoking			
% (SE)	15.4 (0.4)	20.2 (3.3)	-
OR [95% CI]	1.00 (Ref)	1.78 [1.14; 2.78]	0.011

For peer review only - http://bmjopen.bmj.com/site/about/guidelines.xhtml

Daily drinking			
% (SE)	22.8 (0.5)	27.6 (4.2)	-
OR [95% CI]	1.00 (Ref)	1.26 [0.75; 2.13]	0.385
Physical inactivity			
% (SE)	23.0 (0.5)	30.7 (3.7)	-
OR [95% CI]	1.00 (Ref)	1.24 [0.80; 1.92]	0.345

All figures are weighted for sampling probabilities and differential non-response, and are adjusted for age, sex, ethnicity, education, marital status and wealth.
SE = standard error, OR = odds ratio, CI = confidence interval.

For peer review only

History of homelessness and health and wellbeing

Associations between history of homelessness and health and wellbeing are summarised in Table 3. After adjustment for sociodemographics and health behaviours, compared to the group who had not been homeless, the group who had been homeless had 2.49 times higher odds (95% CI 1.54 to 4.03) of reporting a limiting long-standing illness (55.8% vs. 33.5%, $p<0.001$) and 3.30 times higher odds (95% CI 2.01 to 5.42) of depressive symptoms (33.3% vs. 13.0%, $p<0.001$). The group who had been homeless also scored lower on average on measures of life satisfaction (17.34 vs. 19.96, $p<0.001$) and quality of life (39.02 vs. 41.21, $p=0.013$).Rates of fair/bad/very bad self-rated health (41.9% vs. 30.5%) and loneliness (27.1% vs. 21.0%) were higher in the group who had been homeless but these differences did not reach statistical significance (self-rated health $p=0.050$, loneliness $p=0.080$).

Table 3 Associations between history of homelessness and health and wellbeing

	**Had not been homeless**	**Had been homeless**	***p***
Fair/bad/very bad self-rated health			
% (SE)	30.5 (0.6)	41.9 (4.5)	-
OR [95% CI]	1.00 (Ref)	1.63 [1.00; 2.67]	0.050
Limiting long-standing illness			
% (SE)	33.5 (0.6)	55.8 (4.7)	-
OR [95% CI]	1.00 (Ref)	2.49 [1.54; 4.03]	<0.001
Depressive symptoms above threshold			
% (SE)	13.0 (0.4)	33.3 (3.5)	-
OR [95% CI]	1.00 (Ref)	3.30 [2.01; 5.42]	<0.001

For peer review only - http://bmjopen.bmj.com/site/about/guidelines.xhtml

Life satisfaction			
Mean score (SE)	19.96 (0.1)	17.34 (0.7)	-
Coeff. [95% CI]	Ref	-2.78 [-4.18; -1.37]	<0.001
Quality of life			
Mean score (SE)	41.21 (0.1)	39.02 (0.9)	-
Coeff. [95% CI]	Ref	-2.25 [-4.03; -0.47]	0.013
High loneliness			
% (SE)	21.0 (0.5)	27.1 (4.2)	-
OR [95% CI]	1.00 (Ref)	1.56 [0.95; 2.56]	0.080

All figures are weighted for sampling probabilities and differential non-response, and are adjusted for age, sex, ethnicity, education, marital status, wealth, smoking status, alcohol intake and physical activity.
SE = standard error, OR = odds ratio, CI = confidence interval, Coeff = coefficient.
Possible scores on the quality of life scale range from 0-57, and on life satisfaction scale range from 0-30.

DISCUSSION

In the present analyses, a total of 104participants reported having been homeless for one month or more. Those who reported a history of homelessness had a significantly higher odds of smoking than those who had not been homeless and were more likely to drink daily and were inactive but these did not reach significance. Importantly, those who had reported being homeless had a higher odds of reporting limiting long-standing illness and depressive symptoms and scored lower on measures of life satisfaction and quality of life. Taken together, these data suggest that people who transition out of homelessness are at increased risk of partaking in unhealthy behaviour and suffer poorer mental and physical health.

Most smokers find it difficult to quit using tobacco because they are addicted to nicotine.[26] In addition, smoking behaviour is maintained through social networks, such that smokers with social connections who also smoke are less likely to quit.[27] This may be particularly important for people who have been homeless as stigmatised groups may be less able to afford losing the few social connections they have. Considering a very high proportion of homeless people smoke, it is unsurprising that a high proportion continue to smoke when they transition out of homelessness. Moreover, current policies and interventions targeting the homeless tend to focus on behaviours that prevent a successful transition back into society, including drug use and a high alcohol intake, with less emphasis on cigarette smoking. Given the plethora of detrimental consequences of cigarette smoking for both physical and mental health,[28,29] there is a need for targeted smoking cessation interventions for this population.

For peer review only - http://bmjopen.bmj.com/site/about/guidelines.xhtml

The novel finding that people who have ever been homeless are at increased risk of adverse physical and mental health outcomes is important. This suggests that the transition from homelessness is not enough to bring the health profile of this population in alignment with the general public. There are several factors that may account for the observed disparity in health. First, depression is prevalent among the homeless community [30] and is a highly recurrent disorder, [31] thus is likely to reoccur after the transition out of homelessness, with significant personal consequences.[31] Low mood may be partly driving the observed negative associations with life satisfaction, quality of life, and limiting-long standing illness. [32]Interestingly, smoking has been shown to lead to depression,[28] and the present study and others [5–7] have documented particularly high prevalence of smoking in the homeless or ex-homeless population.

Second, people who are homeless are susceptible to multiple health complications. Chronic hepatitis C and co-infections are common among the homeless population.[33] Other conditions that are prevalent among the homeless include tuberculosis, uncontrolled asthma, and dermatologic infestations.[34] These problems are compounded by high rates of drug and alcohol abuse and together likely contribute to limiting-long standing illness and lower quality of life across the lifespan.[35,36]

To our knowledge, this is the first study to examine health outcomes in those who have transitioned out of homelessness. While these findings are important for advancing the evidence base in this area, they should be considered in light of a couple of limitations. The very small number of participants with a history of homelessness in our sample meant that analyses were underpowered to detect modest differences between groups. However, the fact that we observed significant differences in the majority of the outcomes we analysed attests to the strength of these associations. Information on time since the period(s) of homelessness was not available so we were unable to evaluate the extent to which recency of homelessness is related to our outcomes of interest. It is possible that participants who reported a history of homelessness had transitioned out of homelessness many years or even decades prior. If this is the case, it demonstrates the long-lasting impact of homelessness across the lifespan.

CONCLUSION

In conclusion, the present results indicate that older adults in England who have previously been homeless are more likely to engage in lifestyle risk behaviours and have poorer mental and physical health outcomes than those who have never been homeless. Whereas continued initiatives to tackle homelessness itself is important, it is also crucial to consider that even those who have transitioned from homelessness continue

For peer review only - http://bmjopen.bmj.com/site/about/guidelines.xhtml

to be at much higher risk of adverse health behaviours and poor well-being. Therefore, targeted interventions are required to improve health outcomes and quality of life in this population.

Authors Contributions: Study concept and design: LS and SEJ. Analysis and interpretation of data: LS and SEJ. Drafting of the manuscript: All authors. Critical revision of the manuscript for important intellectual content: All authors

Conflicts of Interest: None declared.

Funding: This research did not receive any funding from agencies in the public, commercial, or not-for-profit sectors.

Ethics: Ethical approval was obtained from the National Research Ethics Service and all participants gave full informed consent.

Data sharing: No additional data available.

REFERENCES

1 Fitzpatrick S, Pawson H, Bramley G, *et al.* The homelessness monitor: England 2017. Institute for Social Policy, Environment and Real Estate (I-SPHERE), Heriot-Watt University; City Futures Research Centre, University of New South Wales. London: Crisis 2017.

2 Adebowale V. There is no excuse for homelessness in Britain in 2018. *BMJ* 2018;360:k902. doi:10.1136/bmj.k902

3 Thomson H, Thomas S, Sellstrom E, *et al.* Housing improvements for health and associated socio-economic outcomes. *Cochrane Database Syst Rev* 2013;CD008657. doi:10.1002/14651858.CD008657.pub2

4 Crisis. 2011. *Homelessness: A Silent Killer*. https://www.crisis.org.uk/ending-homelessness/homelessness-knowledge-hub/health-and-wellbeing/homelessness-a-silent-killer-2011/ (accessed 11 Oct 2018).

5 Kermode M, Crofts N, Miller P, *et al.* Health indicators and risks among people experiencing homelessness in Melbourne, 1995-1996. *Aust N Z J Public Health* 1998;22:464–70.

6 Fischer PJ, Breakey WR. The epidemiology of alcohol, drug, and mental disorders among homeless persons. *Am Psychol* 1991;46:1115–28.

For peer review only - http://bmjopen.bmj.com/site/about/guidelines.xhtml

For peer review only

7 Connor SE, Cook RL, Herbert MI, *et al.* Smoking cessation in a homeless population: there is a will, but is there a way? *J Gen Intern Med* 2002;17:369–72.

8 Centers for Disease Control and Prevention (CDC). Cigarette smoking among adults-United States, 2007. *MMWR Morb Mortal Wkly Rep* 2008;57:1221–6.

9 Pedersen P, Christensen AI, Hess U, *et al.* Susydsat-sundhedsproil for social udsatte I Danmark 2007. Copenhagen: Radet for socialt udsatte 2008.

10 O'Toole TP, Gibbon JL, Hanusa BH, *et al.* Self-Reported Changes in Drug and Alcohol Use After Becoming Homeless. *Am J Public Health* 2004;94:830–5. doi:10.2105/AJPH.94.5.830

11 Office of the Deputy Prime Minister. Select Committee on Office of the Deputy Prime Minister: Housing, Planning, Local Government and the Regions Written Evidence: Memorandum by the Office of the Deputy Prime Minister (HOM 53). 2004. https://publications.parliament.uk/pa/cm200304/cmselect/cmodpm/1116/1116we56.htm (accessed 11 Oct 2018).

12 Jones A, Pleace N. Review of single homelessness in the uk 2000-2010. Br. Libr. 2010. http://www.bl.uk/collection-items/review-of-single-homelessness-in-the-uk-20002010 (accessed 11 Oct 2018).

13 Malina RM. Tracking of physical activity and physical fitness across the lifespan. *Res Q Exerc Sport* 1996;67:S48-57.

14 Ben-Shlomo Y, Mishra G, Kuh D. Life Course Epidemiology. In: Ahrens W, Pigeot I, eds. *Handbook of Epidemiology*. New York, NY: Springer New York 2014. 1521–49. doi:10.1007/978-0-387-09834-0_56

15 Steptoe A, Breeze E, Banks J, *et al.* Cohort profile: the English Longitudinal Study of Ageing. *Int J Epidemiol* 2013;42:1640–8. doi:10.1093/ije/dys168

16 Demakakos P, Hamer M, Stamatakis E, *et al.* Low-intensity physical activity is associated with reduced risk of incident type 2 diabetes in older adults: evidence from the English Longitudinal Study of Ageing. *Diabetologia* 2010;53:1877–85. doi:10.1007/s00125-010-1785-x

17 Steptoe A, Jackson SE. The Life Skills of Older Americans: Association with Economic, Psychological, Social, and Health Outcomes. *Sci Rep* 2018;8:9669. doi:10.1038/s41598-018-27909-w

18 DeSalvo KB, Bloser N, Reynolds K, *et al.* Mortality prediction with a single general self-rated health question. A meta-analysis. *J Gen Intern Med* 2006;21:267–75. doi:10.1111/j.1525-1497.2005.00291.x

19 Radloff LS. The CES-D scale. *Appl Psychol Meas* 1977;1:385–401.

20 Steffick DE. Documentation of affective functioning measures in the Health and Retirement Study. 2000.

21 Diener E, Emmons RA, Larsen RJ, *et al.* The satisfaction with life scale. *J Assess* 1985;49:71–75.

For peer review only - http://bmjopen.bmj.com/site/about/guidelines.xhtml

22 Hyde M, Wiggins RD, Higgs P, *et al.* A measure of quality of life in early old age: the theory, development and properties of a needs satisfaction model (CASP-19). *Aging Ment Health* 2003;7:186–194.

23 Russell DW. UCLA Loneliness Scale (Version 3): reliability, validity, and factor structure. *J Pers Assess* 1996;66:20–40. doi:10.1207/s15327752jpa6601_2

24 Steptoe A, Shankar A, Demakakos P, *et al.* Social isolation, loneliness, and all-cause mortality in older men and women. *Proc Natl Acad Sci U S A* 2013;110:5797–801. doi:10.1073/pnas.1219686110

25 Banks J, Karlsen S, Oldfield Z. Socio-economic position. Published Online First: 2003. http://discovery.ucl.ac.uk/15366/1/15366.pdf (accessed 4 Mar 2014).

26 Lynch BS, Bonnie RJ. *The nature of nicotine addiction*. National Academies Press (US) 1994. https://www.ncbi.nlm.nih.gov/books/NBK236759/ (accessed 11 Oct 2018).

27 Christakis NA, Fowler JH. The collective dynamics of smoking in a large social network. *N Engl J Med* 2008;358:2249–58. doi:10.1056/NEJMsa0706154

28 Stubbs B, Vancampfort D, Firth J, *et al.* Association between depression and smoking: A global perspective from 48 low- and middle-income countries. *J Psychiatr Res* 2018;103:142–9. doi:10.1016/j.jpsychires.2018.05.018

29 Sherratt FC, Field JK, Marcus MW. Association between smoking and health outcomes in an economically deprived population: the Liverpool Lung Project. *J Epidemiol Community Health* 2017;71:806–10. doi:10.1136/jech-2016-208730

30 Bassuk EL, Beardslee WR. Depression in homeless mothers: Addressing an unrecognized public health issue. *Am J Orthopsychiatry* 2014;84:73–81. doi:10.1037/h0098949

31 Burcusa SL, Iacono WG. Risk for Recurrence in Depression. *Clin Psychol Rev* 2007;27:959–85. doi:10.1016/j.cpr.2007.02.005

32 Ruo B, Rumsfeld JS, Hlatky MA, *et al.* Depressive Symptoms and Health-Related Quality of Life: The Heart and Soul Study. *JAMA* 2003;290:215–21. doi:10.1001/jama.290.2.215

33 Cheung RC, Hanson AK, Maganti K, *et al.* Viral Hepatitis and Other Infectious Diseases in a Homeless Population. *J Clin Gastroenterol* 2002;34:476-80.

34 Feldmann J, Middleman AB. Homeless adolescents: Common clinical concerns. *Semin Pediatr Infect Dis* 2003;14:6–11. doi:10.1053/spid.2003.127211

35 Spinelli MA, Ponath C, Tieu L, *et al.* Factors associated with substance use in older homeless adults: Results from the HOPE HOME study. *Subst Abuse* 2017;38:88–94. doi:10.1080/08897077.2016.1264534

36 Upshur CC, Jenkins D, Weinreb L, *et al.* Prevalence and predictors of substance use disorders among homeless women seeking primary care: An 11 site survey. *Am J Addict* 2017;26:680–88. doi:10.1111/ajad.12582

For peer review only

For peer review only

For peer review only - http://bmjopen.bmj.com/site/about/guidelines.xhtml

STROBE Statement—Checklist of items that should be included in reports of ***cross-sectional studies***

	Item No	Recommendation	Page No
**Title and abstract**	1	(*a*) Indicate the study's design with a commonly used term in the title or the abstract	1-2
		(*b*) Provide in the abstract an informative and balanced summary of what was done and what was found	2
**Introduction**			
Background/rationale	2	Explain the scientific background and rationale for the investigation being reported	4
Objectives	3	State specific objectives, including any prespecified hypotheses	4-5
**Methods**			
Study design	4	Present key elements of study design early in the paper	5
Setting	5	Describe the setting, locations, and relevant dates, including periods of recruitment, exposure, follow-up, and data collection	5
Participants	6	(*a*) Give the eligibility criteria, and the sources and methods of selection of participants	5
Variables	7	Clearly define all outcomes, exposures, predictors, potential confounders, and effect modifiers. Give diagnostic criteria, if applicable	5-7
Data sources/measurement	8*	For each variable of interest, give sources of data and details of methods of assessment (measurement). Describe comparability of assessment methods if there is more than one group	5-7
Bias	9	Describe any efforts to address potential sources of bias	5-7
Study size	10	Explain how the study size was arrived at	5
Quantitative variables	11	Explain how quantitative variables were handled in the analyses. If applicable, describe which groupings were chosen and why	7-8
Statistical methods	12	(*a*) Describe all statistical methods, including those used to control for confounding	7-8
		(*b*) Describe any methods used to examine subgroups and interactions	7-8
		(*c*) Explain how missing data were addressed	7-8
		(*d*) If applicable, describe analytical methods taking account of sampling strategy	7-8
		(*e*) Describe any sensitivity analyses	7-8
**Results**			
Participants	13*	(a) Report numbers of individuals at each stage of study—eg numbers potentially eligible, examined for eligibility, confirmed eligible, included in the study, completing follow-up, and analysed	8-10
		(b) Give reasons for non-participation at each stage	8-10
		(c) Consider use of a flow diagram	
Descriptive data	14*	(a) Give characteristics of study participants (eg demographic, clinical, social) and information on exposures and potential confounders	8-10
		(b) Indicate number of participants with missing data for each variable of interest	8-10
Outcome data	15*	Report numbers of outcome events or summary measures	8-10
Main results	16	(*a*) Give unadjusted estimates and, if applicable, confounder-adjusted estimates and their precision (eg, 95% confidence interval). Make clear which confounders were adjusted for and why they were included	8-10

For peer review only

		(*b*) Report category boundaries when continuous variables were categorized	8-10
		(*c*) If relevant, consider translating estimates of relative risk into absolute risk for a meaningful time period	8-10
Other analyses	17	Report other analyses done—eg analyses of subgroups and interactions, and sensitivity analyses	8-10
**Discussion**			
Key results	18	Summarise key results with reference to study objectives	11
Limitations	19	Discuss limitations of the study, taking into account sources of potential bias or imprecision. Discuss both direction and magnitude of any potential bias	12
Interpretation	20	Give a cautious overall interpretation of results considering objectives, limitations, multiplicity of analyses, results from similar studies, and other relevant evidence	11-12
Generalisability	21	Discuss the generalisability (external validity) of the study results	11-12
**Other information**			
Funding	22	Give the source of funding and the role of the funders for the present study and, if applicable, for the original study on which the present article is based	13

*Give information separately for exposed and unexposed groups.

Note: An Explanation and Elaboration article discusses each checklist item and gives methodological background and published examples of transparent reporting. The STROBE checklist is best used in conjunction with this article (freely available on the Web sites of PLoS Medicine at http://www.plosmedicine.org/, Annals of Internal Medicine at http://www.annals.org/, and Epidemiology at http://www.epidem.com/). Information on the STROBE Initiative is available at www.strobe-statement.org.

For peer review only

For peer review only - http://bmjopen.bmj.com/site/about/guidelines.xhtml

BMJ Open

Health behaviours and mental and physical health status in older adults with a history of homelessness: a population-based study

Journal:	*BMJ Open*
Manuscript ID	bmjopen-2018-028003.R1
Article Type:	Research
Date Submitted by the Author:	12-Mar-2019
Complete List of Authors:	Smith, Lee; Anglia Ruskin University, Department of Life Sciences<br>Veronese, Nicola; National Research Council, Neuroscience Institute, Aging Branch<br>López-Sánchez, Guillermo Felipe; Faculty of Sport Sciences, University of Murcia<br>Moller, Eloise ; The Single Homeless Project<br>Johnstone, James ; The Cambridge Centre for Sport and Exercise Sciences, Anglia Ruskin University<br>Firth, Joseph ; NICM Health Research Institute, Western Sydney University<br>Grabovac, Igor; Centre for Public Health, Medical University of Vienna, Department of Social and Preventive Medicine<br>Yang, Lin; Medical University of Vienna, Epidemiology<br>Soysal, Pinar; Faculty of Medicine, Bezmialem Vakif University, Department of Geriatric Medicine<br>Jackson, Sarah ; University College London, Department of Behavioural Science and Health
**Primary Subject Heading**:	Epidemiology
Secondary Subject Heading:	Epidemiology
Keywords:	Homelessness, physical health, MENTAL HEALTH, health behaviour, older adults

SCHOLARONE™
Manuscripts

Health behaviours and mental and physical health status in older adults with a history of homelessness: a population-based study

Smith L,[1*] Veronese N,[2] López-Sánchez GF, [3] Moller E,[4] Johnstone J,[5] Firth J,[6] Grabovac I,[7] Yang L,[8] Soysal P,[9] Jackson SE[10*]

1. The Cambridge Centre for Sport and Exercise Sciences, Anglia Ruskin University, Cambridge. Lee.Smith@anglia.ac.uk

2. National Research Council, Neuroscience Institute, Aging Branch, Padova, Italy. ilmannato@gmail.com

3. Faculty of Sport Sciences, University of Murcia, Spain. gfls@um.es

4. The Single Homeless Project, UK, London. Emoller@shp.org.uk

5. The Cambridge Centre for Sport and Exercise Sciences, Anglia Ruskin University, Cambridge. james.johnstone@anglia.ac.uk

6. NICM Health Research Institute, Western Sydney University, Westmead, Australia. j.firth@westernsydney.edu.au

7. Department of Social and Preventive Medicine, Centre for Public Health, Medical University of Vienna, Vienna, Austria. igor.grabovac@meduniwien.ac.at

8. Department of Epidemiology, Center for Public Health, Medical University of Vienna, Vienna, Austria. Lin.yang@muv.ac.at

9. Department of Geriatric Medicine, Faculty of Medicine, Bezmialem Vakif University, Istanbul, Turkey. dr.pinarsoysal@hotmail.com

10. Department of Behavioural Science and Health, University College London, London, UK. s.e.jackson@ucl.ac.uk

***Corresponding authors:** Dr Sarah E Jackson, PhD. Department of Behavioural Science and Health, University College London, 1-19 Torrington Place, London WC1E 6BT s.e.jackson@ucl.ac.uk; and Dr Lee Smith, PhD. Cambridge Centre for Sports and Exercise Sciences, Anglia Ruskin University, Cambridge lee.smith@anglia.ac.uk

Word count (excluding title page, abstract, references, figures and tables): 2726.

For peer review only

For peer review only - http://bmjopen.bmj.com/site/about/guidelines.xhtml

Health behaviours and mental and physical health status in older adults with a history of homelessness: a population-based study

ABSTRACT

Objectives: This study compared (i) levels of engagement in lifestyle risk behaviours and (ii) mental and physical health status in individuals who have previously been homeless to those of individuals who have not. **Design:** Cross-sectional. **Participants:** Data were from participants (n=6,931) of the English Longitudinal Study of Ageing. **Measures:** Participants reported whether they had ever been homeless. We used regression models to analyse associations between homelessness and (i) cigarette smoking, daily alcohol consumption and physical inactivity, adjusting for sociodemographic covariates (age, sex, ethnicity, highest level of education, marital status, and household non-pension wealth), and (ii) self-rated health, limiting long-standing illness, depressive symptoms, life satisfaction, quality of life and loneliness, adjusting for sociodemographics and health behaviours. **Results:** 104 participants (1.5%) reported having been homeless. Individuals who had been homeless were significantly more likely to be physically inactive (OR=1.62, 95% CI 1.44 to 2.52), report fair/bad/very bad self-rated health (OR=1.75, 95% CI 1.07 to 2.86), have a limiting long-standing illness (OR=2.66, 95% CI 1.65 to 4.30) and be depressed (OR=3.06, 95% CI 1.85 to 5.05), and scored lower on measures of life satisfaction (17.34 vs. 19.96, p<0.001) and quality of life (39.02 vs. 41.21, p=0.013). Rates of smoking (20.2% vs. 15.4%, p=0.436), daily drinking (27.6% vs. 22.8%, p=0.385), and loneliness (27.1% vs. 21.0%, p=0.080) were also elevated. **Conclusions:** Those who were once homeless have poorer mental and physical health outcomes and are more likely to be physically inactive. Interventions to improve their health and quality of life are required.

Keywords: Homelessness, physical health, mental health, health behaviour, older adults.

For peer review only - http://bmjopen.bmj.com/site/about/guidelines.xhtml

Strengths and limitations of this study

- To our knowledge, this is the first study to examine health outcomes in those who have transitioned out of homelessness.
- The very small number of participants with a history of homelessness in our sample meant that analyses were underpowered to detect modest differences between groups.
- However, the fact that we observed significant differences in the majority of the outcomes we analysed attests to the strength of these associations.
- Information on time since the period(s) of homelessness was not available so we were unable to evaluate the extent to which recency of homelessness is related to our outcomes of interest.
- Information on what way the participants were homeless (rough sleepers, statutory homeless families) was not available.

For peer review only

For peer review only - http://bmjopen.bmj.com/site/about/guidelines.xhtml

INTRODUCTION

Homelessness is a substantial and growing problem in the United Kingdom. The annual homelessness monitor from Crisis and the Joseph Rowntree Foundation showed that in 2015/16 there were 271,000 local authority homelessness case actions in the UK, a rise of 32% since 2009/10.[1] Being homeless, or at risk of homelessness, has been shown to have a detrimental impact upon mental and physical health.[2] A recent systematic review concluded that people who are homeless are at increased risk of respiratory conditions, depression, anxiety, and excess winter mortality, compared with the general population.[3] Homelessness is associated with premature death, with the single homeless at the highest risk with an average age of death at 47 years, some 30 years lower than in the general population.[4]

Increased morbidity and mortality among the homeless may be driven, at least in part, by higher levels of engagement in lifestyle risk behaviours. Data from the USA indicate that while 19.8% of adult Americans smoke, smoking prevalence is over 70% among those who are homeless.[5–8] Levels of physical activity are also low among the homeless. In a Danish study, approximately 70% of homeless individuals reported no participation in any form of exercise.[9] High levels of alcohol consumption and drug use are also common among this population.[10]

Tackling homelessness is an urgent priority, and targeted policies have been actioned in the UK to rehouse those who are homeless. The Homelessness Directorate was established in 2002 in order to assist local authorities in tackling homelessness.[11] Strategies focus on preventing the need for people to sleep rough in the first place, as well as supporting people to move on from homelessness by helping them to address their needs, improving access to health and substance misuse services, and helping them rebuild their lives through education, training and employment.[12] A number of UK charities (e.g. Crisis, Shelter England, The Single Homeless Project) also work to support people who are homeless in acquiring a home and entering back into employment. With such policies and charities in operation, a significant number of individuals are able to transition out of homelessness.

For peer review only - http://bmjopen.bmj.com/site/about/guidelines.xhtml

While the evidence base on the health risks associated with homelessness is growing, to our knowledge no studies have explored what happens to the health and wellbeing of people when they are no longer homeless. Given that lifestyle behaviours tend to track over the life course,[13] and early life exposures can have a substantial impact on later-life health outcomes,[14] it seems likely that the health risks associated with homelessness may persist, at least to some extent, beyond the period of homelessness. The present study aimed to investigate this through a comparison of (i) levels of engagement in lifestyle risk behaviours and (ii) mental and physical health status in individuals who have previously spent a period of time in their lives as homeless with those of individuals who have never been homeless, in a population-based sample of older adults living in England. Specifically, we aimed to address the following research questions:

1. To what extent do individuals with a history of homelessness differ from those who have never been homeless with regard to smoking status, alcohol intake, and level of physical activity, adjusting for relevant sociodemographic characteristics?
2. To what extent do individuals with a history of homelessness differ from those who have never been homeless with regard to self-rated health, limiting long-standing illness, depressive symptoms, life satisfaction, quality of life, and loneliness, adjusting for relevant sociodemographic characteristics and health behaviours?

We hypothesised that individuals who had previously been homeless would have a higher prevalence of lifestyle risk behaviours and an unfavourable mental and physical health profile compared with those who had never been homeless.

METHODS

Study population

Data were from the English Longitudinal Study of Ageing (ELSA), a nationally-representative longitudinal panel study of men and women aged 50 and older living in households across England.[15] The study began in 2002, with subsequent rounds of data collection at two-year intervals via computer-assisted personal interview and self-completion questionnaires. Wave 3 (2006/07) included a life history questionnaire, which gathered detailed information about important events that occurred in the participants' lives, including whether they had ever been homeless. Of the 9,771 participants interviewed in Wave 3 of ELSA, 7,855

For peer review only - http://bmjopen.bmj.com/site/about/guidelines.xhtml

(80.4%) completed the life history questionnaire. We excluded 924 participants (11.8%) with missing data on homelessness or sociodemographic covariates, leaving a final sample for analysis of 6,931 men and women. Ethical approval was obtained from the National Research Ethics Service and all participants gave full informed consent.

Patient and Public Involvement

Patients were not involved in the design of this study.

Measures

History of homelessness

Participants were asked whether they had ever been homeless for one month or more (yes/no).

Health behaviours

Smoking status was assessed with the question *"Do you smoke cigarettes at all nowadays"* (yes/no).

Frequency of alcohol intake over the past 12 months was reported on an 8-point scale from "not at all in the last 12 months" to "almost every day". We dichotomised responses to distinguish between participants drinking almost every day ("daily drinking") vs. less than this.

Physical activity was assessed with three items that asked respondents about the frequency with which they took part in vigorous, moderate and low-intensity activities (more than once a week, once a week, 1-3 times a month, hardly ever/never)[16]. Responses were dichotomised as follows: inactive (no moderate/vigorous activity on a weekly basis) vs. active (moderate or vigorous activity at least once a week).

Health and wellbeing

Self-rated health was assessed using a single item: *"Would you say your health is… very good/good/fair/bad/very bad?"* We analysed the proportion of individuals rating their health as fair, bad or very bad, as is commonly done in analyses of this variable.[17,18]

Limiting long-standing illness was assessed with two questions: (1) *"Do you have any long-standing illness, disability, or infirmity? By long-standing I mean anything that has troubled you over a period of time or that*

For peer review only - http://bmjopen.bmj.com/site/about/guidelines.xhtml

is likely to affect you over a period of time." If they responded yes, they were asked (2) *"Does this illness or disability limit your activities in any way?"* Affirmation of a long-standing illness and any form of limitation classified the participant as having a limiting long-standing illness.

Depressive symptoms were assessed with an eight-item version of the Center for Epidemiologic Studies Depression Scale (CES-D) [19], a scale highly validated for use in older adults [20]. This asks about feelings over the last week (e.g. "Over the last week have you felt sad"), with binary response options (1=yes, 0=no). Positively framed items were reverse scored. Data were dichotomised using an established cut-off, with a score of 4 or higher indicating significant symptomatology.[20]

Life satisfaction was assessed with the Satisfaction With Life Scale,[21] which asks respondents to rate the extent to which they agree with five statements: "*In most ways my life is close to my ideal*"; "*The conditions of my life are excellent*"; "*I am satisfied with my life*"; "*So far I have got the important things I want in life*"; "*If I could live my life again, I would change almost nothing*" on a scale from 0 (strongly disagree) to 6 (strongly agree). Responses are summed to produce a total score between 0 and 30, with higher scores indicating greater life satisfaction.

Quality of life was assessed with the CASP-19,[22] a scale designed to measure quality of life in older people. Items cover four domains of quality of life; control (e.g. "*I feel that what happens to me is out of my control*"), autonomy (e.g. "*My health stops me from doing things I want to do*"), self-realisation (e.g. "*I feel that life is full of opportunities*"), and pleasure (e.g. "*I enjoy being in the company of others*"). Respondents are asked how often each statement applies to them (often=0, sometimes=1, not often=2, never=3). Positively-worded items are reverse scored so that a higher total score indicates higher quality of life (range: 0–57).

Loneliness was measured using a three-item short form of the Revised University of California Los Angeles (UCLA) Loneliness Scale.[23] Participants were asked: "*How often do you feel you lack companionship?*"(hardly ever or never=1, some of the time=2, often=3). Scores ranged from 3 to 9, with higher scores indicating greater loneliness. They were dichotomised at ≥6 versus <6 to indicate high versus low loneliness.[24]

Sociodemographic covariates

For peer review only - http://bmjopen.bmj.com/site/about/guidelines.xhtml

Interviewers collected information on age, sex, ethnicity, the highest level of education, marital status and wealth. For these analyses, ethnicity was categorised as white or non-white. We classified education as low (no formal qualifications), intermediate (up to degree) or high (degree or higher). Marital status was categorised as married or unmarried (never married, divorced or widowed). Wealth was categorised into five equal groups of net total non-pension wealth measured at the benefit unit level (a benefit unit is a couple or single person along with any dependent children they might have) across all ELSA participants who took part in Wave 3. Wealth has been identified as a particularly appropriate indicator of SES in this age group.[25]

Statistical analysis

All analyses were conducted using SPSS version 24. Data were weighted to correct for sampling probabilities and for differential non-response and to calibrate back to the 2011 National Census population distributions for age and sex. The weights accounted for the differential probability of being included in Wave 3 of ELSA and for non-response to the life history interview.

Differences in sociodemographic characteristics of the groups who did and did not report a history of homelessness were tested using independent t-tests for continuous variables and Pearson's chi-square tests for categorical variables. We used binary logistic regression to analyse associations between history of homelessness and cigarette smoking, daily drinking and physical inactivity, adjusting for sociodemographic covariates. We then used linear regression (for continuous outcomes) and binary logistic regression (for categorical outcomes) to analyse associations between history of homelessness and health and wellbeing, adjusting for sociodemographics and health behaviours. In all models, the reference category was the group without a history of homelessness. A *p*-value <0.05 was used to indicate statistical significance.

RESULTS

Sample characteristics

Of the 6,931 participants in our sample, 104 (1.5%) reported having been homeless for one month or more and 6,827 (98.5%) had never been homeless for one month or more. Sample characteristics in relation to history of homelessness are summarised in Table 1. On average, participants who had been homeless were

For peer review only - http://bmjopen.bmj.com/site/about/guidelines.xhtml

significantly younger than those who had not been homeless (60.9 vs. 65.7 years) and a greater proportion were non-white (6.6% vs. 3.1%), unmarried (54.3% vs. 33.8%) and from the lowest quintile of wealth (44.3% vs. 18.9%). A marginally higher proportion of the group who had been homeless were male (53.8% vs. 46.7%) although the difference was not statistically significant (*p*=0.149). There was no significant difference between groups in the highest level of education achieved.

Table 1 Sample characteristics in relation to history of homelessness

	**Had not been homeless (*n*=6,827)**[1]	**Had been homeless (*n*=104)**	***p***
Age (years), mean (SD)	65.74 (10.64)	60.94 (8.32)	<0.001
Sex			
Men	46.7	53.8	0.149
Women	53.3	46.2	-
Ethnicity			
White	96.9	93.4	0.042
Non-white	3.1	6.6	-
Highest level of education			
No qualifications	32.4	27.4	0.541
Below degree	52.2	56.6	-
Degree or higher	15.5	16.0	-
Marital status			
Married	66.2	45.7	<0.001
Unmarried	33.8	54.3	-
Wealth quintile			
1 (poorest)	18.9	44.3	<0.001
2	19.5	13.2	-
3	20.6	14.2	-
4	20.0	16.0	-
5 (richest)	21.0	12.3	-

[1] Unweighted sample sizes.
All figures are weighted for sampling probabilities and differential non-response.
Values are percentages unless otherwise stated.
SD = standard deviation.

For peer review only - http://bmjopen.bmj.com/site/about/guidelines.xhtml

History of homelessness and health behaviours

Associations between history of homelessness and health behaviours are shown in Table 2. After adjustment for age, sex, ethnicity, education, marital status and wealth, participants who had been homeless had 1.62 times higher odds (95% CI 1.44 to 2.52) of being inactive than those who had not been homeless (30.7% vs. 23.0%, *p*=0.031). Rates of smoking (20.2% vs. 15.4%) and daily drinking (27.6% vs. 22.8%) were also higher in the group who had been homeless, but differences were not statistically significant.

Table 2 Associations between history of homelessness and health behaviours

	**Had not been homeless**	**Had been homeless**	***p***
Smoking			
% (SE)	15.4 (0.4)	20.2 (3.3)	-
OR [95% CI]	1.00 (Ref)	1.21 [0.75; 1.94]	0.436
Daily drinking			
% (SE)	22.8 (0.5)	27.6 (4.2)	-
OR [95% CI]	1.00 (Ref)	1.31 [0.77; 2.21]	0.321
Physical inactivity			
% (SE)	23.0 (0.5)	30.7 (3.7)	-
OR [95% CI]	1.00 (Ref)	1.62 [1.04; 2.52]	0.031

All figures are weighted for sampling probabilities and differential non-response, and are adjusted for age, sex, ethnicity, education, marital status and wealth.
SE = standard error, OR = odds ratio, CI = confidence interval.

History of homelessness and health and wellbeing

Associations between history of homelessness and health and wellbeing are summarised in Table 3. After adjustment for sociodemographics and health behaviours, compared with the group who had not been homeless, the group who had been homeless had 1.75 times higher odds (95% CI 1.07 to 2.86) of reporting

For peer review only - http://bmjopen.bmj.com/site/about/guidelines.xhtml

For peer review only

fair/bad/very bad self-rated health (41.9% vs. 30.5%, *p*=0.025), 2.66 times higher odds (95% CI 1.65 to 4.30) of reporting a limiting long-standing illness (55.8% vs. 33.5%, *p*<0.001) and 3.06 times higher odds (95% CI 1.85 to 5.05) of depressive symptoms (33.3% vs. 13.0%, *p*<0.001). The group who had been homeless also scored lower on average on measures of life satisfaction (17.34 vs. 19.96, *p*<0.001) and quality of life (39.02 vs. 41.21, *p*=0.013). The rate of loneliness (27.1% vs. 21.0%) was higher in the group who had been homeless but this difference did not reach statistical significance (*p*=0.110).

Table 3 Associations between history of homelessness and health and wellbeing

	**Had not been homeless**	**Had been homeless**	***p***
Fair/bad/very bad self-rated health			
% (SE)	30.5 (0.6)	41.9 (4.5)	-
OR [95% CI]	1.00 (Ref)	1.75 [1.07; 2.86]	0.025
Limiting long-standing illness			
% (SE)	33.5 (0.6)	55.8 (4.7)	-
OR [95% CI]	1.00 (Ref)	2.66 [1.65; 4.30]	<0.001
Depressive symptoms above threshold			
% (SE)	13.0 (0.4)	33.3 (3.5)	-
OR [95% CI]	1.00 (Ref)	3.06 [1.85; 5.05]	<0.001
Life satisfaction			
Mean score (SE)	19.96 (0.1)	17.34 (0.7)	-
Coeff. [95% CI]	Ref	-2.78 [-4.18; -1.37]	<0.001
Quality of life			
Mean score (SE)	41.21 (0.1)	39.02 (0.9)	-
Coeff. [95% CI]	Ref	-2.25 [-4.03; -0.47]	0.013
High loneliness			
% (SE)	21.0 (0.5)	27.1 (4.2)	-
OR [95% CI]	1.00 (Ref)	1.50 [0.91; 2.47]	0.110

All figures are weighted for sampling probabilities and differential non-response and adjusted for age, sex, ethnicity, education, marital status, wealth, smoking status, alcohol intake and physical activity.
SE = standard error, OR = odds ratio, CI = confidence interval, Coeff = coefficient.
Possible scores on the quality of life scale range from 0-57, and on life satisfaction scale range from 0-30.

DISCUSSION

In the present analyses, a total of 104 participants reported having been homeless for one month or more. Those who reported a history of homelessness had significantly higher odds of physical inactivity than those

For peer review only - http://bmjopen.bmj.com/site/about/guidelines.xhtml

who had not been homeless and were more likely to smoke and drink daily but these did not reach significance. Importantly, those who had reported being homeless had a higher odds of reporting fair/bad/very bad self-rated health, limiting long-standing illness and depressive symptoms and scored lower on measures of life satisfaction and quality of life. Taken together, these data suggest that people who transition out of homelessness may be at increased risk of partaking in unhealthy behaviour and suffer poorer mental and physical health.

The finding that those who were previously homeless were more likely to be inactive than those who were not is of importance. Indeed, sustained and regular participation in physical activity can aid in the prevention against, and improve the profile of, non-communicable diseases – including those in relation to both physical (e.g. cardio respiratory; [26]) and mental (e.g. anxiety and depression; [27,28]) health, both of which are common in homeless populations [3]. Moreover, similar health profiles were observed in the present manuscript in a population who has transitioned from homelessness. Literature suggests that levels of physical activity track across the life course [29]. Importantly, those who are homeless have critically low levels of physical activity. For example, in a Danish study, approximately 70% of the homeless reported no participation in any form of exercise [9]. This low level of physical activity is potentially tracking through the transition from homelessness.

The novel finding that people who have ever been homeless are at increased risk of adverse physical and mental health outcomes is important. This suggests that the transition from homelessness is not enough to bring the health profile of this population in alignment with the general public. There are several factors that may account for the observed disparity in health. First, depression is prevalent among the homeless community [30] and is a highly recurrent disorder, [31] thus is likely to reoccur after the transition out of homelessness, with significant personal consequences.[31] Low mood may be partly driving the observed negative associations with life satisfaction, quality of life, and limiting-long standing illness. [32]

Second, people who are homeless are susceptible to multiple health complications. Chronic hepatitis C and co-infections are common among the homeless population.[33] Other conditions that are prevalent among the homeless include tuberculosis, uncontrolled asthma, and dermatologic infestations.[34] These problems are compounded by high rates of drug and alcohol abuse and together likely contribute to poorer self-rated health, limiting-long standing illness and lower quality of life across the lifespan.[35,36]

To our knowledge, this is the first study to examine health outcomes in those who have transitioned out of homelessness. While these findings are important for advancing the evidence base in this area, they should

For peer review only - http://bmjopen.bmj.com/site/about/guidelines.xhtml

be considered in light of a couple of limitations. The very small number of participants with a history of homelessness in our sample meant that analyses were underpowered to detect modest differences between groups. However, the fact that we observed significant differences in the majority of the outcomes we analysed attests to the strength of these associations. Nevertheless, future research using larger samples is required to confirm or refute our findings. While we adjusted for a wide range of sociodemographic and behavioural covariates, it is possible that the results could be explained by residual confounding by unmeasured variables – i.e. the group reporting a history of homelessness were deprived in ways that were not reflected in the existing variables. Information on time since the period(s) of homelessness was not available so we were unable to evaluate the extent to which recency of homelessness is related to our outcomes of interest. It is possible that participants who reported a history of homelessness had transitioned out of homelessness many years or even decades prior. In addition, information on type of homelessness was not available. It is therefore unknown whether those who reported once being homeless were "rough sleepers" or "statutory homeless". Type of homelessness may have varying influences on health/ behaviour and future research to tease out the influence of type of previous homelessness on these outcomes is required. Finally, ELSA does not collect data on those currently homeless and thus it was not possible to have a "currently homeless" category in the present analyses. Given that we did not observe significant differences in some outcomes previously demonstrated to differ between currently homeless and housed populations (e.g. smoking), it might be the case that those who manage to transition out of homelessness are able to offset some of the increased risk associated with having been homeless. Future research may wish to compare those never homeless, those currently homeless, and those previously homeless to gain a deeper insight.

CONCLUSION

In conclusion, the present results indicate that older adults in England who have previously been homeless are more likely to be physically inactive and have poorer mental and physical health outcomes than those who have never been homeless.

Continued initiatives to tackle homelessness itself is important. It is also crucial to consider that even those who have transitioned from homelessness continue to be at much higher risk of poor health and wellbeing. Therefore, continued monitoring and targeted interventions are required to improve health outcomes and quality of life in this population. Such interventions may wish to consider lifestyle risk behaviours to improve mental and physical health status.

For peer review only

For peer review only - http://bmjopen.bmj.com/site/about/guidelines.xhtml

Authors Contributions: Study concept and design: LS and SEJ. Analysis and interpretation of data: LS and SEJ. Drafting of the manuscript: LS, NV, GFLS, EM, JJ, JF, IG, LY, PS, SEJ. Critical revision of the manuscript for important intellectual content: LS, NV, GFLS, EM, JJ, JF, IG, LY, PS, SEJ.

Conflicts of Interest: None declared.

Funding: This research did not receive any funding from agencies in the public, commercial, or not-for-profit sectors.

Ethics: Ethical approval was obtained from the National Research Ethics Service and all participants gave full informed consent.

Data sharing: No additional data available.

REFERENCES

1 Fitzpatrick S, Pawson H, Bramley G, *et al.* The homelessness monitor: England 2017. Institute for Social Policy, Environment and Real Estate (I-SPHERE), Heriot-Watt University; City Futures Research Centre, University of New South Wales 2017.

2 Adebowale V. There is no excuse for homelessness in Britain in 2018. *BMJ* 2018;**360**:k902. doi:10.1136/bmj.k902

3 Thomson H, Thomas S, Sellstrom E, *et al.* Housing improvements for health and associated socio-economic outcomes. *Cochrane Database Syst Rev* 2013;:CD008657. doi:10.1002/14651858.CD008657.pub2

4 Homelessness: A Silent Killer (2011). Crisis. https://www.crisis.org.uk/ending-homelessness/homelessness-knowledge-hub/health-and-wellbeing/homelessness-a-silent-killer-2011/ (accessed 11 Oct 2018).

5 Kermode M, Crofts N, Miller P, *et al.* Health indicators and risks among people experiencing homelessness in Melbourne, 1995-1996. *Aust N Z J Public Health* 1998;**22**:464–70.

6 Fischer PJ, Breakey WR. The epidemiology of alcohol, drug, and mental disorders among homeless persons. *Am Psychol* 1991;**46**:1115–28.

7 Connor SE, Cook RL, Herbert MI, *et al.* Smoking cessation in a homeless population: there is a will, but is there a way? *J Gen Intern Med* 2002;**17**:369–72.

8 Centers for Disease Control and Prevention (CDC). Cigarette smoking among adults--United States, 2007. *MMWR Morb Mortal Wkly Rep* 2008;**57**:1221–6.

For peer review only - http://bmjopen.bmj.com/site/about/guidelines.xhtml

9 Pedersen P, Christensen AI, Hess U, *et al.* Susydsat-sundhedsproil for social udsatte I Danmark 2007. Copenhagen: : Radet for socialt udsatte 2008.

10 O'Toole TP, Gibbon JL, Hanusa BH, *et al.* Self-Reported Changes in Drug and Alcohol Use After Becoming Homeless. *Am J Public Health* 2004;**94**:830–5. doi:10.2105/AJPH.94.5.830

11 Office of the Deputy Prime Minister. Select Committee on Office of the Deputy Prime Minister: Housing, Planning, Local Government and the Regions Written Evidence: Memorandum by the Office of the Deputy Prime Minister (HOM 53). 2004.https://publications.parliament.uk/pa/cm200304/cmselect/cmodpm/1116/1116we56.htm (accessed 11 Oct 2018).

12 Jones A, Pleace N. Review of single homelessness in the uk 2000-2010. Br. Libr. 2010.http://www.bl.uk/collection-items/review-of-single-homelessness-in-the-uk-20002010 (accessed 11 Oct 2018).

13 Malina RM. Tracking of physical activity and physical fitness across the lifespan. *Res Q Exerc Sport* 1996;**67**:S48-57.

14 Ben-Shlomo Y, Mishra G, Kuh D. Life Course Epidemiology. In: Ahrens W, Pigeot I, eds. *Handbook of Epidemiology*. New York, NY: : Springer New York 2014. 1521–49. doi:10.1007/978-0-387-09834-0_56

15 Steptoe A, Breeze E, Banks J, *et al.* Cohort profile: the English Longitudinal Study of Ageing. *Int J Epidemiol* 2013;**42**:1640–8. doi:10.1093/ije/dys168

16 Demakakos P, Hamer M, Stamatakis E, *et al.* Low-intensity physical activity is associated with reduced risk of incident type 2 diabetes in older adults: evidence from the English Longitudinal Study of Ageing. *Diabetologia* 2010;**53**:1877–85. doi:10.1007/s00125-010-1785-x

17 Steptoe A, Jackson SE. The Life Skills of Older Americans: Association with Economic, Psychological, Social, and Health Outcomes. *Sci Rep* 2018;**8**:9669. doi:10.1038/s41598-018-27909-w

18 DeSalvo KB, Bloser N, Reynolds K, *et al.* Mortality prediction with a single general self-rated health question. A meta-analysis. *J Gen Intern Med* 2006;**21**:267–75. doi:10.1111/j.1525-1497.2005.00291.x

19 Radloff LS. The CES-D scale. *Appl Psychol Meas* 1977;**1**:385–401.

20 Steffick DE. Documentation of affective functioning measures in the Health and Retirement Study. 2000.

21 Diener E, Emmons RA, Larsen RJ, *et al.* The satisfaction with life scale. *J Assess* 1985;**49**:71–75.

22 Hyde M, Wiggins RD, Higgs P, *et al.* A measure of quality of life in early old age: the theory, development and properties of a needs satisfaction model (CASP-19). *Aging Ment Health* 2003;**7**:186–194.

23 Russell DW. UCLA Loneliness Scale (Version 3): reliability, validity, and factor structure. *J Pers Assess* 1996;**66**:20–40. doi:10.1207/s15327752jpa6601_2

For peer review only - http://bmjopen.bmj.com/site/about/guidelines.xhtml

24 Steptoe A, Shankar A, Demakakos P, *et al.* Social isolation, loneliness, and all-cause mortality in older men and women. *Proc Natl Acad Sci U S A* 2013;**110**:5797–801. doi:10.1073/pnas.1219686110

25 Banks J, Karlsen S, Oldfield Z. Socio-economic position. Published Online First: 2003.http://discovery.ucl.ac.uk/15366/1/15366.pdf (accessed 4 Mar 2014).

26 Li J, Siegrist J. Physical activity and risk of cardiovascular disease--a meta-analysis of prospective cohort studies. *Int J Environ Res Public Health* 2012;**9**:391–407. doi:10.3390/ijerph9020391

27 Schuch FB, Vancampfort D, Richards J, *et al.* Exercise as a treatment for depression: A meta-analysis adjusting for publication bias. *J Psychiatr Res* 2016;**77**:42–51. doi:10.1016/j.jpsychires.2016.02.023

28 Stonerock GL, Hoffman BM, Smith PJ, *et al.* Exercise as Treatment for Anxiety: Systematic Review and Analysis. *Ann Behav Med Publ Soc Behav Med* 2015;**49**:542–56. doi:10.1007/s12160-014-9685-9

29 Smith L, Gardner B, Aggio D, *et al.* Association between participation in outdoor play and sport at 10years old with physical activity in adulthood. *Prev Med* 2015;**74**:31–5. doi:10.1016/j.ypmed.2015.02.004

30 Bassuk EL, Beardslee WR. Depression in homeless mothers: Addressing an unrecognized public health issue. *Am J Orthopsychiatry* 2014;**84**:73–81. doi:10.1037/h0098949

31 Burcusa SL, Iacono WG. Risk for Recurrence in Depression. *Clin Psychol Rev* 2007;**27**:959–85. doi:10.1016/j.cpr.2007.02.005

32 Ruo B, Rumsfeld JS, Hlatky MA, *et al.* Depressive Symptoms and Health-Related Quality of Life: The Heart and Soul Study. *JAMA* 2003;**290**:215–21. doi:10.1001/jama.290.2.215

33 Cheung RC, Hanson AK, Maganti K, *et al.* Viral Hepatitis and Other Infectious Diseases in a Homeless Population. *J Clin Gastroenterol* 2002;**34**:476.

34 Feldmann J, Middleman AB. Homeless adolescents: Common clinical concerns. *Semin Pediatr Infect Dis* 2003;**14**:6–11. doi:10.1053/spid.2003.127211

35 Spinelli MA, Ponath C, Tieu L, *et al.* Factors associated with substance use in older homeless adults: Results from the HOPE HOME study. *Subst Abuse* 2017;**38**:88–94. doi:10.1080/08897077.2016.1264534

36 Upshur CC, Jenkins D, Weinreb L, *et al.* Prevalence and predictors of substance use disorders among homeless women seeking primary care: An 11 site survey. *Am J Addict* 2017;**26**:680–8. doi:10.1111/ajad.12582

For peer review only

For peer review only - http://bmjopen.bmj.com/site/about/guidelines.xhtml

STROBE Statement—Checklist of items that should be included in reports of ***cross-sectional studies***

	Item No	Recommendation	Page No
**Title and abstract**	1	(*a*) Indicate the study's design with a commonly used term in the title or the abstract	1-2
		(*b*) Provide in the abstract an informative and balanced summary of what was done and what was found	2
**Introduction**			
Background/rationale	2	Explain the scientific background and rationale for the investigation being reported	4
Objectives	3	State specific objectives, including any prespecified hypotheses	4-5
**Methods**			
Study design	4	Present key elements of study design early in the paper	5
Setting	5	Describe the setting, locations, and relevant dates, including periods of recruitment, exposure, follow-up, and data collection	5
Participants	6	(*a*) Give the eligibility criteria, and the sources and methods of selection of participants	5
Variables	7	Clearly define all outcomes, exposures, predictors, potential confounders, and effect modifiers. Give diagnostic criteria, if applicable	5-7
Data sources/measurement	8*	For each variable of interest, give sources of data and details of methods of assessment (measurement). Describe comparability of assessment methods if there is more than one group	5-7
Bias	9	Describe any efforts to address potential sources of bias	5-7
Study size	10	Explain how the study size was arrived at	5
Quantitative variables	11	Explain how quantitative variables were handled in the analyses. If applicable, describe which groupings were chosen and why	7-8
Statistical methods	12	(*a*) Describe all statistical methods, including those used to control for confounding	7-8
		(*b*) Describe any methods used to examine subgroups and interactions	7-8
		(*c*) Explain how missing data were addressed	7-8
		(*d*) If applicable, describe analytical methods taking account of sampling strategy	7-8
		(*e*) Describe any sensitivity analyses	7-8
**Results**			
Participants	13*	(a) Report numbers of individuals at each stage of study—eg numbers potentially eligible, examined for eligibility, confirmed eligible, included in the study, completing follow-up, and analysed	8-10
		(b) Give reasons for non-participation at each stage	8-10
		(c) Consider use of a flow diagram	
Descriptive data	14*	(a) Give characteristics of study participants (eg demographic, clinical, social) and information on exposures and potential confounders	8-10
		(b) Indicate number of participants with missing data for each variable of interest	8-10
Outcome data	15*	Report numbers of outcome events or summary measures	8-10
Main results	16	(*a*) Give unadjusted estimates and, if applicable, confounder-adjusted estimates and their precision (eg, 95% confidence interval). Make clear which confounders were adjusted for and why they were included	8-10

For peer review only

		(*b*) Report category boundaries when continuous variables were categorized	8-10
		(*c*) If relevant, consider translating estimates of relative risk into absolute risk for a meaningful time period	8-10
Other analyses	17	Report other analyses done—eg analyses of subgroups and interactions, and sensitivity analyses	8-10
**Discussion**			
Key results	18	Summarise key results with reference to study objectives	11
Limitations	19	Discuss limitations of the study, taking into account sources of potential bias or imprecision. Discuss both direction and magnitude of any potential bias	12
Interpretation	20	Give a cautious overall interpretation of results considering objectives, limitations, multiplicity of analyses, results from similar studies, and other relevant evidence	11-12
Generalisability	21	Discuss the generalisability (external validity) of the study results	11-12
**Other information**			
Funding	22	Give the source of funding and the role of the funders for the present study and, if applicable, for the original study on which the present article is based	13

*Give information separately for exposed and unexposed groups.

Note: An Explanation and Elaboration article discusses each checklist item and gives methodological background and published examples of transparent reporting. The STROBE checklist is best used in conjunction with this article (freely available on the Web sites of PLoS Medicine at http://www.plosmedicine.org/, Annals of Internal Medicine at http://www.annals.org/, and Epidemiology at http://www.epidem.com/). Information on the STROBE Initiative is available at www.strobe-statement.org.

For peer review only

BMJ Open

Health behaviours and mental and physical health status in older adults with a history of homelessness: a cross-sectional population-based study in England

Journal:	*BMJ Open*
Manuscript ID	bmjopen-2018-028003.R2
Article Type:	Research
Date Submitted by the Author:	19-Mar-2019
Complete List of Authors:	Smith, Lee; Anglia Ruskin University, Department of Life Sciences<br>Veronese, Nicola; National Research Council, Neuroscience Institute, Aging Branch<br>López-Sánchez, Guillermo Felipe; Faculty of Sport Sciences, University of Murcia<br>Moller, Eloise ; The Single Homeless Project<br>Johnstone, James ; The Cambridge Centre for Sport and Exercise Sciences, Anglia Ruskin University<br>Firth, Joseph ; NICM Health Research Institute, Western Sydney University<br>Grabovac, Igor; Centre for Public Health, Medical University of Vienna, Department of Social and Preventive Medicine<br>Yang, Lin; Medical University of Vienna, Epidemiology<br>Soysal, Pinar; Faculty of Medicine, Bezmialem Vakif University, Department of Geriatric Medicine<br>Jackson, Sarah ; University College London, Department of Behavioural Science and Health
**Primary Subject Heading**:	Epidemiology
Secondary Subject Heading:	Epidemiology
Keywords:	Homelessness, physical health, MENTAL HEALTH, health behaviour, older adults

SCHOLARONE™
Manuscripts

Health behaviours and mental and physical health status in older adults with a history of homelessness: a cross-sectional population-based study in England

Smith L,[1*] Veronese N,[2] López-Sánchez GF, [3] Moller E,[4] Johnstone J,[5] Firth J,[6] Grabovac I,[7] Yang L,[8] Soysal P,[9] Jackson SE[10*]

1. The Cambridge Centre for Sport and Exercise Sciences, Anglia Ruskin University, Cambridge. Lee.Smith@anglia.ac.uk

2. National Research Council, Neuroscience Institute, Aging Branch, Padova, Italy. ilmannato@gmail.com

3. Faculty of Sport Sciences, University of Murcia, Spain. gfls@um.es

4. The Single Homeless Project, UK, London. Emoller@shp.org.uk

5. The Cambridge Centre for Sport and Exercise Sciences, Anglia Ruskin University, Cambridge. james.johnstone@anglia.ac.uk

6. NICM Health Research Institute, Western Sydney University, Westmead, Australia. j.firth@westernsydney.edu.au

7. Department of Social and Preventive Medicine, Centre for Public Health, Medical University of Vienna, Vienna, Austria. igor.grabovac@meduniwien.ac.at

8. Department of Epidemiology, Center for Public Health, Medical University of Vienna, Vienna, Austria. Lin.yang@muv.ac.at

9. Department of Geriatric Medicine, Faculty of Medicine, Bezmialem Vakif University, Istanbul, Turkey. dr.pinarsoysal@hotmail.com

10. Department of Behavioural Science and Health, University College London, London, UK. s.e.jackson@ucl.ac.uk

***Corresponding authors:** Dr Sarah E Jackson, PhD. Department of Behavioural Science and Health, University College London, 1-19 Torrington Place, London WC1E 6BT s.e.jackson@ucl.ac.uk; and Dr Lee Smith, PhD. Cambridge Centre for Sports and Exercise Sciences, Anglia Ruskin University, Cambridge lee.smith@anglia.ac.uk

Word count (excluding title page, abstract, references, figures and tables): 2726.

For peer review only - http://bmjopen.bmj.com/site/about/guidelines.xhtml

Health behaviours and mental and physical health status in older adults with a history of homelessness: a cross-sectional population-based study in England

ABSTRACT

Objectives: This study compared (i) levels of engagement in lifestyle risk behaviours and (ii) mental and physical health status in individuals who have previously been homeless to those of individuals who have not. **Design:** Cross-sectional. **Participants:** Data were from participants (n=6,931) of the English Longitudinal Study of Ageing. **Measures:** Participants reported whether they had ever been homeless. We used regression models to analyse associations between homelessness and (i) cigarette smoking, daily alcohol consumption and physical inactivity, adjusting for sociodemographic covariates (age, sex, ethnicity, highest level of education, marital status, and household non-pension wealth), and (ii) self-rated health, limiting long-standing illness, depressive symptoms, life satisfaction, quality of life and loneliness, adjusting for sociodemographics and health behaviours. **Results:** 104 participants (1.5%) reported having been homeless. Individuals who had been homeless were significantly more likely to be physically inactive (OR=1.62, 95% CI 1.44 to 2.52), report fair/bad/very bad self-rated health (OR=1.75, 95% CI 1.07 to 2.86), have a limiting long-standing illness (OR=2.66, 95% CI 1.65 to 4.30) and be depressed (OR=3.06, 95% CI 1.85 to 5.05), and scored lower on measures of life satisfaction (17.34 vs. 19.96, p<0.001) and quality of life (39.02 vs. 41.21, p=0.013). Rates of smoking (20.2% vs. 15.4%, p=0.436), daily drinking (27.6% vs. 22.8%, p=0.385), and loneliness (27.1% vs. 21.0%, p=0.080) were also elevated. **Conclusions:** Those who were once homeless have poorer mental and physical health outcomes and are more likely to be physically inactive. Interventions to improve their health and quality of life are required.

Keywords: Homelessness, physical health, mental health, health behaviour, older adults.

For peer review only - http://bmjopen.bmj.com/site/about/guidelines.xhtml

Strengths and limitations of this study

- To our knowledge, this is the first study to examine health outcomes in those who have transitioned out of homelessness.
- The very small number of participants with a history of homelessness in our sample meant that analyses were underpowered to detect modest differences between groups.
- However, the fact that we observed significant differences in the majority of the outcomes we analysed attests to the strength of these associations.
- Information on time since the period(s) of homelessness was not available so we were unable to evaluate the extent to which recency of homelessness is related to our outcomes of interest.
- Information on what way the participants were homeless (rough sleepers, statutory homeless families) was not available.

For peer review only

For peer review only - http://bmjopen.bmj.com/site/about/guidelines.xhtml

INTRODUCTION

Homelessness is a substantial and growing problem in the United Kingdom. The annual homelessness monitor from Crisis and the Joseph Rowntree Foundation showed that in 2015/16 there were 271,000 local authority homelessness case actions in the UK, a rise of 32% since 2009/10.[1] Being homeless, or at risk of homelessness, has been shown to have a detrimental impact upon mental and physical health.[2] A recent systematic review concluded that people who are homeless are at increased risk of respiratory conditions, depression, anxiety, and excess winter mortality, compared with the general population.[3] Homelessness is associated with premature death, with the single homeless at the highest risk with an average age of death at 47 years, some 30 years lower than in the general population.[4] Moreover, the standardised mortality ratios reported for the homeless vary between studies and countries but are typically 2–5 times the age-standardised general population.[5]

Increased morbidity and mortality among the homeless may be driven, at least in part, by higher levels of engagement in lifestyle risk behaviours. Data from the USA indicate that while 19.8% of adult Americans smoke, smoking prevalence is over 70% among those who are homeless.[6–9] Levels of physical activity are also low among the homeless. In a Danish study, approximately 70% of homeless individuals reported no participation in any form of exercise.[10] High levels of alcohol consumption and drug use are also common among this population.[11]

Tackling homelessness is an urgent priority, and targeted policies have been actioned in the UK to rehouse those who are homeless. The Homelessness Directorate was established in 2002 in order to assist local authorities in tackling homelessness.[12] Strategies focus on preventing the need for people to sleep rough in the first place, as well as supporting people to move on from homelessness by helping them to address their needs, improving access to health and substance misuse services, and helping them rebuild their lives through education, training and employment.[13] A number of UK charities (e.g. Crisis, Shelter England, The Single Homeless Project) also work to support people who are homeless in acquiring a home and entering

For peer review only - http://bmjopen.bmj.com/site/about/guidelines.xhtml

back into employment. With such policies and charities in operation, a significant number of individuals are able to transition out of homelessness.

While the evidence base on the health risks associated with homelessness is growing, to our knowledge no studies have explored what happens to the health and wellbeing of people when they are no longer homeless. Given that lifestyle behaviours tend to track over the life course,[14] and early life exposures can have a substantial impact on later-life health outcomes,[15] it seems likely that the health risks associated with homelessness may persist, at least to some extent, beyond the period of homelessness. The present study aimed to investigate this through a comparison of (i) levels of engagement in lifestyle risk behaviours and (ii) mental and physical health status in individuals who have previously spent a period of time in their lives as homeless with those of individuals who have never been homeless, in a population-based sample of older adults living in England. Specifically, we aimed to address the following research questions:

1. To what extent do individuals with a history of homelessness differ from those who have never been homeless with regard to smoking status, alcohol intake, and level of physical activity, adjusting for relevant sociodemographic characteristics?
2. To what extent do individuals with a history of homelessness differ from those who have never been homeless with regard to self-rated health, limiting long-standing illness, depressive symptoms, life satisfaction, quality of life, and loneliness, adjusting for relevant sociodemographic characteristics and health behaviours?

We hypothesised that individuals who had previously been homeless would have a higher prevalence of lifestyle risk behaviours and an unfavourable mental and physical health profile compared with those who had never been homeless.

METHODS

Study population

Data were from the English Longitudinal Study of Ageing (ELSA), a nationally-representative longitudinal panel study of men and women aged 50 and older living in households across England.[16] The study began in 2002, with subsequent rounds of data collection at two-year intervals via computer-assisted personal

For peer review only - http://bmjopen.bmj.com/site/about/guidelines.xhtml

interview and self-completion questionnaires. Wave 3 (2006/07) included a life history questionnaire, which gathered detailed information about important events that occurred in the participants' lives, including whether they had ever been homeless. Of the 9,771 participants interviewed in Wave 3 of ELSA, 7,855 (80.4%) completed the life history questionnaire. We excluded 924 participants (11.8%) with missing data on homelessness or sociodemographic covariates, leaving a final sample for analysis of 6,931 men and women. Ethical approval was obtained from the National Research Ethics Service and all participants gave full informed consent.

Patient and Public Involvement

Patients were not involved in the design of this study.

Measures

History of homelessness

Participants were asked whether they had ever been homeless for one month or more (yes/no).

Health behaviours

Smoking status was assessed with the question "*Do you smoke cigarettes at all nowadays"* (yes/no).

Frequency of alcohol intake over the past 12 months was reported on an 8-point scale from "not at all in the last 12 months" to "almost every day". We dichotomised responses to distinguish between participants drinking almost every day ("daily drinking") vs. less than this.

Physical activity was assessed with three items that asked respondents about the frequency with which they took part in vigorous, moderate and low-intensity activities (more than once a week, once a week, 1-3 times a month, hardly ever/never)[17]. Responses were dichotomised as follows: inactive (no moderate/vigorous activity on a weekly basis) vs. active (moderate or vigorous activity at least once a week).

Health and wellbeing

For peer review only - http://bmjopen.bmj.com/site/about/guidelines.xhtml

Self-rated health was assessed using a single item: *"Would you say your health is… very good/good/fair/bad/very bad?"* We analysed the proportion of individuals rating their health as fair, bad or very bad, as is commonly done in analyses of this variable.[18,19]

Limiting long-standing illness was assessed with two questions: (1) *"Do you have any long-standing illness, disability, or infirmity? By long-standing I mean anything that has troubled you over a period of time or that is likely to affect you over a period of time."* If they responded yes, they were asked (2) *"Does this illness or disability limit your activities in any way?"* Affirmation of a long-standing illness and any form of limitation classified the participant as having a limiting long-standing illness.

Depressive symptoms were assessed with an eight-item version of the Center for Epidemiologic Studies Depression Scale (CES-D) [20], a scale highly validated for use in older adults [21]. This asks about feelings over the last week (e.g. "Over the last week have you felt sad"), with binary response options (1=yes, 0=no). Positively framed items were reverse scored. Data were dichotomised using an established cut-off, with a score of 4 or higher indicating significant symptomatology.[21]

Life satisfaction was assessed with the Satisfaction With Life Scale,[22] which asks respondents to rate the extent to which they agree with five statements: "*In most ways my life is close to my ideal*"; "*The conditions of my life are excellent*"; "*I am satisfied with my life*"; "*So far I have got the important things I want in life*"; "*If I could live my life again, I would change almost nothing*" on a scale from 0 (strongly disagree) to 6 (strongly agree). Responses are summed to produce a total score between 0 and 30, with higher scores indicating greater life satisfaction.

Quality of life was assessed with the CASP-19,[23] a scale designed to measure quality of life in older people. Items cover four domains of quality of life; control (e.g. "*I feel that what happens to me is out of my control*"), autonomy (e.g. "*My health stops me from doing things I want to do*"), self-realisation (e.g. "*I feel that life is full of opportunities*"), and pleasure (e.g. "*I enjoy being in the company of others*"). Respondents are asked how often each statement applies to them (often=0, sometimes=1, not often=2, never=3). Positively-worded items are reverse scored so that a higher total score indicates higher quality of life (range: 0–57).

Loneliness was measured using a three-item short form of the Revised University of California Los Angeles (UCLA) Loneliness Scale.[24] Participants were asked: "*How often do you feel you lack companionship?*"(hardly ever or never=1, some of the time=2, often=3). Scores ranged from 3 to 9, with

For peer review only - http://bmjopen.bmj.com/site/about/guidelines.xhtml

higher scores indicating greater loneliness. They were dichotomised at ≥6 versus <6 to indicate high versus low loneliness.[25]

Sociodemographic covariates

Interviewers collected information on age, sex, ethnicity, the highest level of education, marital status and wealth. For these analyses, ethnicity was categorised as white or non-white. We classified education as low (no formal qualifications), intermediate (up to degree) or high (degree or higher). Marital status was categorised as married or unmarried (never married, divorced or widowed). Wealth was categorised into five equal groups of net total non-pension wealth measured at the benefit unit level (a benefit unit is a couple or single person along with any dependent children they might have) across all ELSA participants who took part in Wave 3. Wealth has been identified as a particularly appropriate indicator of SES in this age group.[26]

Statistical analysis

All analyses were conducted using SPSS version 24. Data were weighted to correct for sampling probabilities and for differential non-response and to calibrate back to the 2011 National Census population distributions for age and sex. The weights accounted for the differential probability of being included in Wave 3 of ELSA and for non-response to the life history interview.

Differences in sociodemographic characteristics of the groups who did and did not report a history of homelessness were tested using independent t-tests for continuous variables and Pearson's chi-square tests for categorical variables. We used binary logistic regression to analyse associations between history of homelessness and cigarette smoking, daily drinking and physical inactivity, adjusting for sociodemographic covariates. We then used linear regression (for continuous outcomes) and binary logistic regression (for categorical outcomes) to analyse associations between history of homelessness and health and wellbeing, adjusting for sociodemographics and health behaviours. In all models, the reference category was the group without a history of homelessness. A *p*-value <0.05 was used to indicate statistical significance.

RESULTS

For peer review only - http://bmjopen.bmj.com/site/about/guidelines.xhtml

Sample characteristics

Of the 6,931 participants in our sample, 104 (1.5%) reported having been homeless for one month or more and 6,827 (98.5%) had never been homeless for one month or more. Sample characteristics in relation to history of homelessness are summarised in Table 1. On average, participants who had been homeless were significantly younger than those who had not been homeless (60.9 vs. 65.7 years) and a greater proportion were non-white (6.6% vs. 3.1%), unmarried (54.3% vs. 33.8%) and from the lowest quintile of wealth (44.3% vs. 18.9%). A marginally higher proportion of the group who had been homeless were male (53.8% vs. 46.7%) although the difference was not statistically significant (p=0.149). There was no significant difference between groups in the highest level of education achieved.

Table 1 Sample characteristics in relation to history of homelessness

	**Had not been homeless (*n*=6,827)[1]**	**Had been homeless (*n*=104)**	***p***
Age (years), mean (SD)	65.74 (10.64)	60.94 (8.32)	<0.001
Sex			
Men	46.7	53.8	0.149
Women	53.3	46.2	-
Ethnicity			
White	96.9	93.4	0.042
Non-white	3.1	6.6	-
Highest level of education			
No qualifications	32.4	27.4	0.541
Below degree	52.2	56.6	-
Degree or higher	15.5	16.0	-
Marital status			
Married	66.2	45.7	<0.001
Unmarried	33.8	54.3	-
Wealth quintile			
1 (poorest)	18.9	44.3	<0.001

For peer review only - http://bmjopen.bmj.com/site/about/guidelines.xhtml

2	19.5	13.2	-
3	20.6	14.2	-
4	20.0	16.0	-
5 (richest)	21.0	12.3	-

[1] Unweighted sample sizes.
All figures are weighted for sampling probabilities and differential non-response.
Values are percentages unless otherwise stated.
SD = standard deviation.

History of homelessness and health behaviours

Associations between history of homelessness and health behaviours are shown in Table 2. After adjustment for age, sex, ethnicity, education, marital status and wealth, participants who had been homeless had 1.62 times higher odds (95% CI 1.44 to 2.52) of being inactive than those who had not been homeless (30.7% vs. 23.0%, p=0.031). Rates of smoking (20.2% vs. 15.4%) and daily drinking (27.6% vs. 22.8%) were also higher in the group who had been homeless, but differences were not statistically significant.

Table 2 Associations between history of homelessness and health behaviours

	Had not been homeless	Had been homeless	*p*
Smoking			
% (SE)	15.4 (0.4)	20.2 (3.3)	-
OR [95% CI]	1.00 (Ref)	1.21 [0.75; 1.94]	0.436
Daily drinking			
% (SE)	22.8 (0.5)	27.6 (4.2)	-
OR [95% CI]	1.00 (Ref)	1.31 [0.77; 2.21]	0.321
Physical inactivity			
% (SE)	23.0 (0.5)	30.7 (3.7)	-
OR [95% CI]	1.00 (Ref)	1.62 [1.04; 2.52]	0.031

All figures are weighted for sampling probabilities and differential non-response, and are adjusted for age, sex, ethnicity, education, marital status and wealth.
SE = standard error, OR = odds ratio, CI = confidence interval.

For peer review only - http://bmjopen.bmj.com/site/about/guidelines.xhtml

History of homelessness and health and wellbeing

Associations between history of homelessness and health and wellbeing are summarised in Table 3. After adjustment for sociodemographics and health behaviours, compared with the group who had not been homeless, the group who had been homeless had 1.75 times higher odds (95% CI 1.07 to 2.86) of reporting fair/bad/very bad self-rated health (41.9% vs. 30.5%, p=0.025), 2.66 times higher odds (95% CI 1.65 to 4.30) of reporting a limiting long-standing illness (55.8% vs. 33.5%, p<0.001) and 3.06 times higher odds (95% CI 1.85 to 5.05) of depressive symptoms (33.3% vs. 13.0%, p<0.001). The group who had been homeless also scored lower on average on measures of life satisfaction (17.34 vs. 19.96, p<0.001) and quality of life (39.02 vs. 41.21, p=0.013). The rate of loneliness (27.1% vs. 21.0%) was higher in the group who had been homeless but this difference did not reach statistical significance (p=0.110).

Table 3 Associations between history of homelessness and health and wellbeing

	**Had not been homeless**	**Had been homeless**	***p***
Fair/bad/very bad self-rated health			
% (SE)	30.5 (0.6)	41.9 (4.5)	-
OR [95% CI]	1.00 (Ref)	1.75 [1.07; 2.86]	0.025
Limiting long-standing illness			
% (SE)	33.5 (0.6)	55.8 (4.7)	-
OR [95% CI]	1.00 (Ref)	2.66 [1.65; 4.30]	<0.001
Depressive symptoms above threshold			
% (SE)	13.0 (0.4)	33.3 (3.5)	-
OR [95% CI]	1.00 (Ref)	3.06 [1.85; 5.05]	<0.001
Life satisfaction			
Mean score (SE)	19.96 (0.1)	17.34 (0.7)	-
Coeff. [95% CI]	Ref	-2.78 [-4.18; -1.37]	<0.001
Quality of life			
Mean score (SE)	41.21 (0.1)	39.02 (0.9)	-
Coeff. [95% CI]	Ref	-2.25 [-4.03; -0.47]	0.013
High loneliness			
% (SE)	21.0 (0.5)	27.1 (4.2)	-
OR [95% CI]	1.00 (Ref)	1.50 [0.91; 2.47]	0.110

All figures are weighted for sampling probabilities and differential non-response and adjusted for age, sex, ethnicity, education, marital status, wealth, smoking status, alcohol intake and physical activity.
SE = standard error, OR = odds ratio, CI = confidence interval, Coeff = coefficient.
Possible scores on the quality of life scale range from 0-57, and on life satisfaction scale range from 0-30.

For peer review only

For peer review only - http://bmjopen.bmj.com/site/about/guidelines.xhtml

DISCUSSION

In the present analyses, a total of 104 participants reported having been homeless for one month or more. Those who reported a history of homelessness had significantly higher odds of physical inactivity than those who had not been homeless and were more likely to smoke and drink daily but these did not reach significance. Importantly, those who had reported being homeless had a higher odds of reporting fair/bad/very bad self-rated health, limiting long-standing illness and depressive symptoms and scored lower on measures of life satisfaction and quality of life. Taken together, these data suggest that people who transition out of homelessness may be at increased risk of partaking in unhealthy behaviour and suffer poorer mental and physical health.

The finding that those who were previously homeless were more likely to be inactive than those who were not is of importance. Indeed, sustained and regular participation in physical activity can aid in the prevention against, and improve the profile of, non-communicable diseases – including those in relation to both physical (e.g. cardio respiratory; [27]) and mental (e.g. anxiety and depression; [28,29]) health, both of which are common in homeless populations [3]. Moreover, similar health profiles were observed in the present manuscript in a population who has transitioned from homelessness. Literature suggests that levels of physical activity track across the life course [30]. Importantly, those who are homeless have critically low levels of physical activity. For example, in a Danish study, approximately 70% of the homeless reported no participation in any form of exercise [10]. This low level of physical activity is potentially tracking through the transition from homelessness.

The novel finding that people who have ever been homeless are at increased risk of adverse physical and mental health outcomes is important. This suggests that the transition from homelessness is not enough to bring the health profile of this population in alignment with the general public. There are several factors that may account for the observed disparity in health. First, depression is prevalent among the homeless community [31] and is a highly recurrent disorder, [32] thus is likely to reoccur after the transition out of homelessness, with significant personal consequences.[32] Low mood may be partly driving the observed negative associations with life satisfaction, quality of life, and limiting-long standing illness. [33]

Second, people who are homeless are susceptible to multiple health complications. Chronic hepatitis C and co-infections are common among the homeless population.[34] Other conditions that are prevalent among

For peer review only - http://bmjopen.bmj.com/site/about/guidelines.xhtml

the homeless include tuberculosis, uncontrolled asthma, and dermatologic infestations.[35] These problems are compounded by high rates of drug and alcohol abuse and together likely contribute to poorer self-rated health, limiting-long standing illness and lower quality of life across the lifespan.[36,37]

Interestingly, while differences in health outcomes (self-rated health, limiting long-standing illness, depressive symptoms, life satisfaction and QOL) between the present sample (ex homeless) and those who have not been homeless were significant, the magnitude of the associations was smaller than has been documented in previous studies [38]. This may be owing to a degree of 'recovery' from homelessness. It may also be an artefact of the type of homelessness. The majority of the present sample who had experienced homelessness may have been "statutory homeless" where health outcomes are likely better than rough sleeping.

To our knowledge, this is the first study to examine health outcomes in those who have transitioned out of homelessness. While these findings are important for advancing the evidence base in this area, they should be considered in light of a couple of limitations. The very small number of participants with a history of homelessness in our sample meant that analyses were underpowered to detect modest differences between groups. However, the fact that we observed significant differences in the majority of the outcomes we analysed attests to the strength of these associations. Nevertheless, future research using larger samples is required to confirm or refute our findings. While we adjusted for a wide range of sociodemographic and behavioural covariates, it is possible that the results could be explained by residual confounding by unmeasured variables – i.e. the group reporting a history of homelessness were deprived in ways that were not reflected in the existing variables. Information on time since the period(s) of homelessness was not available so we were unable to evaluate the extent to which recency of homelessness is related to our outcomes of interest. It is possible that participants who reported a history of homelessness had transitioned out of homelessness many years or even decades prior. In addition, information on type of homelessness was not available. It is therefore unknown whether those who reported once being homeless were "statutory homeless", lived on the streets, stayed in a shelter, abandoned building or vehicle, etc. Type of homelessness may have varying influences on health and behaviour. It is plausible to assume that those who are rough sleepers (living on streets, abandoned buildings or vehicles) are at a higher risk of poor health, for example, owing to exposure to cold weather and wet conditions or lack of access to essential facilities such as bathrooms. However, those who are rough sleepers are much more likely to be male (86% male) [1] and a relatively large proportion of our sample who were once homeless were female (46.7%). It may be that the present sample are not representative of the

For peer review only - http://bmjopen.bmj.com/site/about/guidelines.xhtml

wider homeless population (or at least rough sleepers) in the UK. Future research to tease out the influence of type of previous homelessness on health/ behaviour outcomes is required. Finally, ELSA does not collect data on those currently homeless and thus it was not possible to have a "currently homeless" category in the present analyses. Given that we did not observe significant differences in some outcomes previously demonstrated to differ between currently homeless and housed populations (e.g. smoking), it might be the case that those who manage to transition out of homelessness are able to offset some of the increased risk associated with having been homeless. Future research may wish to compare those never homeless, those currently homeless, and those previously homeless to gain a deeper insight.

CONCLUSION

In conclusion, the present results indicate that older adults in England who have previously been homeless are more likely to be physically inactive and have poorer mental and physical health outcomes than those who have never been homeless.

Continued initiatives to tackle homelessness itself is important. It is also crucial to consider that even those who have transitioned from homelessness continue to be at much higher risk of poor health and wellbeing. Therefore, continued monitoring and targeted interventions are required to improve health outcomes and quality of life in this population. Such interventions may wish to consider lifestyle risk behaviours to improve mental and physical health status.

Authors Contributions: Study concept and design: LS and SEJ. Analysis and interpretation of data: LS and SEJ. Drafting of the manuscript: LS, NV, GFLS, EM, JJ, JF, IG, LY, PS, SEJ. Critical revision of the manuscript for important intellectual content: LS, NV, GFLS, EM, JJ, JF, IG, LY, PS, SEJ.

Conflicts of Interest: None declared.

Funding: This research did not receive any funding from agencies in the public, commercial, or not-for-profit sectors.

Ethics: Ethical approval was obtained from the National Research Ethics Service and all participants gave full informed consent.

For peer review only - http://bmjopen.bmj.com/site/about/guidelines.xhtml

For peer review only

Data sharing: No additional data available.

REFERENCES

1 Fitzpatrick S, Pawson H, Bramley G, *et al.* The homelessness monitor: England 2017. Institute for Social Policy, Environment and Real Estate (I-SPHERE), Heriot-Watt University; City Futures Research Centre, University of New South Wales 2017.

2 Adebowale V. There is no excuse for homelessness in Britain in 2018. *BMJ* 2018;**360**:k902. doi:10.1136/bmj.k902

3 Thomson H, Thomas S, Sellstrom E, *et al.* Housing improvements for health and associated socio-economic outcomes. *Cochrane Database Syst Rev* 2013;:CD008657. doi:10.1002/14651858.CD008657.pub2

4 Homelessness: A Silent Killer (2011). Crisis. https://www.crisis.org.uk/ending-homelessness/homelessness-knowledge-hub/health-and-wellbeing/homelessness-a-silent-killer-2011/ (accessed 11 Oct 2018).

5 Fazel S, Geddes JR, Kushel M. The health of homeless people in high-income countries: descriptive epidemiology, health consequences, and clinical and policy recommendations. *The Lancet* 2014;**384**:1529–40. doi:10.1016/S0140-6736(14)61132-6

6 Kermode M, Crofts N, Miller P, *et al.* Health indicators and risks among people experiencing homelessness in Melbourne, 1995-1996. *Aust N Z J Public Health* 1998;**22**:464–70.

7 Fischer PJ, Breakey WR. The epidemiology of alcohol, drug, and mental disorders among homeless persons. *Am Psychol* 1991;**46**:1115–28.

8 Connor SE, Cook RL, Herbert MI, *et al.* Smoking cessation in a homeless population: there is a will, but is there a way? *J Gen Intern Med* 2002;**17**:369–72.

9 Centers for Disease Control and Prevention (CDC). Cigarette smoking among adults--United States, 2007. *MMWR Morb Mortal Wkly Rep* 2008;**57**:1221–6.

10 Pedersen P, Christensen AI, Hess U, *et al.* Susydsat-sundhedsproil for social udsatte I Danmark 2007. Copenhagen: : Radet for socialt udsatte 2008.

11 O'Toole TP, Gibbon JL, Hanusa BH, *et al.* Self-Reported Changes in Drug and Alcohol Use After Becoming Homeless. *Am J Public Health* 2004;**94**:830–5. doi:10.2105/AJPH.94.5.830

12 Office of the Deputy Prime Minister. Select Committee on Office of the Deputy Prime Minister: Housing, Planning, Local Government and the Regions Written Evidence: Memorandum by the Office of the Deputy Prime Minister (HOM 53). 2004.https://publications.parliament.uk/pa/cm200304/cmselect/cmodpm/1116/1116we56.htm (accessed 11 Oct 2018).

For peer review only - http://bmjopen.bmj.com/site/about/guidelines.xhtml

13 Jones A, Pleace N. Review of single homelessness in the uk 2000-2010. Br. Libr. 2010.http://www.bl.uk/collection-items/review-of-single-homelessness-in-the-uk-20002010 (accessed 11 Oct 2018).

14 Malina RM. Tracking of physical activity and physical fitness across the lifespan. *Res Q Exerc Sport* 1996;**67**:S48-57.

15 Ben-Shlomo Y, Mishra G, Kuh D. Life Course Epidemiology. In: Ahrens W, Pigeot I, eds. *Handbook of Epidemiology*. New York, NY: : Springer New York 2014. 1521–49. doi:10.1007/978-0-387-09834-0_56

16 Steptoe A, Breeze E, Banks J, *et al.* Cohort profile: the English Longitudinal Study of Ageing. *Int J Epidemiol* 2013;**42**:1640–8. doi:10.1093/ije/dys168

17 Demakakos P, Hamer M, Stamatakis E, *et al.* Low-intensity physical activity is associated with reduced risk of incident type 2 diabetes in older adults: evidence from the English Longitudinal Study of Ageing. *Diabetologia* 2010;**53**:1877–85. doi:10.1007/s00125-010-1785-x

18 Steptoe A, Jackson SE. The Life Skills of Older Americans: Association with Economic, Psychological, Social, and Health Outcomes. *Sci Rep* 2018;**8**:9669. doi:10.1038/s41598-018-27909-w

19 DeSalvo KB, Bloser N, Reynolds K, *et al.* Mortality prediction with a single general self-rated health question. A meta-analysis. *J Gen Intern Med* 2006;**21**:267–75. doi:10.1111/j.1525-1497.2005.00291.x

20 Radloff LS. The CES-D scale. *Appl Psychol Meas* 1977;**1**:385–401.

21 Steffick DE. Documentation of affective functioning measures in the Health and Retirement Study. 2000.

22 Diener E, Emmons RA, Larsen RJ, *et al.* The satisfaction with life scale. *J Assess* 1985;**49**:71–75.

23 Hyde M, Wiggins RD, Higgs P, *et al.* A measure of quality of life in early old age: the theory, development and properties of a needs satisfaction model (CASP-19). *Aging Ment Health* 2003;**7**:186–194.

24 Russell DW. UCLA Loneliness Scale (Version 3): reliability, validity, and factor structure. *J Pers Assess* 1996;**66**:20–40. doi:10.1207/s15327752jpa6601_2

25 Steptoe A, Shankar A, Demakakos P, *et al.* Social isolation, loneliness, and all-cause mortality in older men and women. *Proc Natl Acad Sci U S A* 2013;**110**:5797–801. doi:10.1073/pnas.1219686110

26 Banks J, Karlsen S, Oldfield Z. Socio-economic position. Published Online First: 2003.http://discovery.ucl.ac.uk/15366/1/15366.pdf (accessed 4 Mar 2014).

27 Li J, Siegrist J. Physical activity and risk of cardiovascular disease--a meta-analysis of prospective cohort studies. *Int J Environ Res Public Health* 2012;**9**:391–407. doi:10.3390/ijerph9020391

28 Schuch FB, Vancampfort D, Richards J, *et al.* Exercise as a treatment for depression: A meta-analysis adjusting for publication bias. *J Psychiatr Res* 2016;**77**:42–51. doi:10.1016/j.jpsychires.2016.02.023

For peer review only - http://bmjopen.bmj.com/site/about/guidelines.xhtml

29 Stonerock GL, Hoffman BM, Smith PJ, *et al.* Exercise as Treatment for Anxiety: Systematic Review and Analysis. *Ann Behav Med Publ Soc Behav Med* 2015;**49**:542–56. doi:10.1007/s12160-014-9685-9

30 Smith L, Gardner B, Aggio D, *et al.* Association between participation in outdoor play and sport at 10years old with physical activity in adulthood. *Prev Med* 2015;**74**:31–5. doi:10.1016/j.ypmed.2015.02.004

31 Bassuk EL, Beardslee WR. Depression in homeless mothers: Addressing an unrecognized public health issue. *Am J Orthopsychiatry* 2014;**84**:73–81. doi:10.1037/h0098949

32 Burcusa SL, Iacono WG. Risk for Recurrence in Depression. *Clin Psychol Rev* 2007;**27**:959–85. doi:10.1016/j.cpr.2007.02.005

33 Ruo B, Rumsfeld JS, Hlatky MA, *et al.* Depressive Symptoms and Health-Related Quality of Life: The Heart and Soul Study. *JAMA* 2003;**290**:215–21. doi:10.1001/jama.290.2.215

34 Cheung RC, Hanson AK, Maganti K, *et al.* Viral Hepatitis and Other Infectious Diseases in a Homeless Population. *J Clin Gastroenterol* 2002;**34**:476.

35 Feldmann J, Middleman AB. Homeless adolescents: Common clinical concerns. *Semin Pediatr Infect Dis* 2003;**14**:6–11. doi:10.1053/spid.2003.127211

36 Spinelli MA, Ponath C, Tieu L, *et al.* Factors associated with substance use in older homeless adults: Results from the HOPE HOME study. *Subst Abuse* 2017;**38**:88–94. doi:10.1080/08897077.2016.1264534

37 Upshur CC, Jenkins D, Weinreb L, *et al.* Prevalence and predictors of substance use disorders among homeless women seeking primary care: An 11 site survey. *Am J Addict* 2017;**26**:680–8. doi:10.1111/ajad.12582

38 Munoz RT, Hellman CM, Buster B, *et al.* Life Satisfaction, Hope, and Positive Emotions as Antecedents of Health Related Quality of Life Among Homeless Individuals. *Int J Appl Posit Psychol* 2016;**1**:69–89. doi:10.1007/s41042-017-0005-z

For peer review only

For peer review only - http://bmjopen.bmj.com/site/about/guidelines.xhtml

STROBE Statement—Checklist of items that should be included in reports of ***cross-sectional studies***

	Item No	Recommendation	Page No
**Title and abstract**	1	(*a*) Indicate the study's design with a commonly used term in the title or the abstract	1-2
		(*b*) Provide in the abstract an informative and balanced summary of what was done and what was found	2
**Introduction**			
Background/rationale	2	Explain the scientific background and rationale for the investigation being reported	4
Objectives	3	State specific objectives, including any prespecified hypotheses	4-5
**Methods**			
Study design	4	Present key elements of study design early in the paper	5
Setting	5	Describe the setting, locations, and relevant dates, including periods of recruitment, exposure, follow-up, and data collection	5
Participants	6	(*a*) Give the eligibility criteria, and the sources and methods of selection of participants	5
Variables	7	Clearly define all outcomes, exposures, predictors, potential confounders, and effect modifiers. Give diagnostic criteria, if applicable	5-7
Data sources/measurement	8*	For each variable of interest, give sources of data and details of methods of assessment (measurement). Describe comparability of assessment methods if there is more than one group	5-7
Bias	9	Describe any efforts to address potential sources of bias	5-7
Study size	10	Explain how the study size was arrived at	5
Quantitative variables	11	Explain how quantitative variables were handled in the analyses. If applicable, describe which groupings were chosen and why	7-8
Statistical methods	12	(*a*) Describe all statistical methods, including those used to control for confounding	7-8
		(*b*) Describe any methods used to examine subgroups and interactions	7-8
		(*c*) Explain how missing data were addressed	7-8
		(*d*) If applicable, describe analytical methods taking account of sampling strategy	7-8
		(*e*) Describe any sensitivity analyses	7-8
**Results**			
Participants	13*	(a) Report numbers of individuals at each stage of study—eg numbers potentially eligible, examined for eligibility, confirmed eligible, included in the study, completing follow-up, and analysed	8-10
		(b) Give reasons for non-participation at each stage	8-10
		(c) Consider use of a flow diagram	
Descriptive data	14*	(a) Give characteristics of study participants (eg demographic, clinical, social) and information on exposures and potential confounders	8-10
		(b) Indicate number of participants with missing data for each variable of interest	8-10
Outcome data	15*	Report numbers of outcome events or summary measures	8-10
Main results	16	(*a*) Give unadjusted estimates and, if applicable, confounder-adjusted estimates and their precision (eg, 95% confidence interval). Make clear which confounders were adjusted for and why they were included	8-10

For peer review only - http://bmjopen.bmj.com/site/about/guidelines.xhtml

		(*b*) Report category boundaries when continuous variables were categorized	8-10
		(*c*) If relevant, consider translating estimates of relative risk into absolute risk for a meaningful time period	8-10
Other analyses	17	Report other analyses done—eg analyses of subgroups and interactions, and sensitivity analyses	8-10
**Discussion**			
Key results	18	Summarise key results with reference to study objectives	11
Limitations	19	Discuss limitations of the study, taking into account sources of potential bias or imprecision. Discuss both direction and magnitude of any potential bias	12
Interpretation	20	Give a cautious overall interpretation of results considering objectives, limitations, multiplicity of analyses, results from similar studies, and other relevant evidence	11-12
Generalisability	21	Discuss the generalisability (external validity) of the study results	11-12
**Other information**			
Funding	22	Give the source of funding and the role of the funders for the present study and, if applicable, for the original study on which the present article is based	13

*Give information separately for exposed and unexposed groups.

Note: An Explanation and Elaboration article discusses each checklist item and gives methodological background and published examples of transparent reporting. The STROBE checklist is best used in conjunction with this article (freely available on the Web sites of PLoS Medicine at http://www.plosmedicine.org/, Annals of Internal Medicine at http://www.annals.org/, and Epidemiology at http://www.epidem.com/). Information on the STROBE Initiative is available at www.strobe-statement.org.

For peer review only

For peer review only - http://bmjopen.bmj.com/site/about/guidelines.xhtml
